# Imperfect Maturation of Erythroid Progenitors in Patients with Cirrhosis-Associated Anemia

**DOI:** 10.3390/cimb48050511

**Published:** 2026-05-14

**Authors:** Deepika Lal, Jaswinder Singh Maras, Rakhi Maiwall, Anupam Kumar, Chhagan Bihari

**Affiliations:** 1 Department of Molecular and Cellular Medicine, Institute of Liver and Biliary Sciences, New Delhi 110070, India; ddeepika18021@gmail.com (D.L.); jassi2param@gmail.com (J.S.M.); 2 Department of Hepatology, Institute of Liver and Biliary Sciences, New Delhi 110070, India; rakhi_2011@yahoo.co.in; 3 Department of Pathology, Institute of Liver and Biliary Sciences, New Delhi 110070, India

**Keywords:** anemia, chronic liver disease, erythropoiesis, hemoglobin, inflammation

## Abstract

**Background and Aims**: Anemia is seen in nearly >70% of patients with cirrhosis and is often non-responsive to nutritional supplements; therefore, we assessed the erythropoiesis and associated alteration in bone marrow (BM). **Methods**: It is a cross-sectional study. Flow cytometry was performed to assess the hematopoietic stem cells (HSCs) and erythroid population of 60 patients with cirrhosis compared with patients with 7 non-cirrhotic portal fibrosis (NCPF) and 3 controls. Proteomics were performed of the pure CD71 erythroid population taken from patients with cirrhosis to decipher the internal abnormalities supported by validation experiments. Real Time PCR, colony assay and heme quantification, cytokine array, and ELISA were performed to assess erythropoietic stimulating agents (ESA), inflammatory cytokines, and growth factors as an external factor affecting erythropoiesis. **Results**: We found a decrease in intermediate erythroid progenitors [IEPs; CD71+ CD235a+], conversely early erythroid precursors [EEP; CD71+ CD235a−] and late erythroid progenitors [LEP; CD71− CD235a+] were increased (*p* < 0.05) in cirrhotic and NCPF as compared to control. However, unlike NCPF, cirrhosis exhibited decreased CD71+ transferrin receptor (TfR1) expression over erythroid cells and increased immature erythrocytes (*p* < 0.05) in peripheral circulation. In vitro culture of erythroid precursors showed impaired differentiation and maturation that was confirmed by the reduced (*p* < 0.05) number of erythroid colonies (BFU-E). Proteomics analysis showed downregulated proteins associated with hemoglobin synthesis, ROS detoxification, translation, and mitochondrial activity. Furthermore, we found an altered expression of genes related to erythropoiesis and hemoglobin synthesis and increase (*p* < 0.05) in inflammatory cytokines such as IL-5, TRAIL-R2, TGF-α, and TGF-β in BM. **Conclusions**: This study suggests that the dysregulated erythropoiesis observed in patients with cirrhosis having anemia is maintained despite adequate nutrition.

## 1. Introduction

Anemia is a common complication in CLD (chronic liver disease); up to 75% of patients with cirrhosis have reported reduced hemoglobin (Hb) levels [1]. Occurrence of anemia is associated with complex and multifactorial etiologies and includes portal hypertension-induced sequestration, alteration in erythropoietin (EPO), BM suppression, and increased blood loss (e.g., hemorrhage, hemolysis) [2,3]. In most cases of CLD, the type of anemia encountered is normocytic normochromic anemia, attributable to the chronic inflammatory state [4]. Patients with cirrhosis may not respond to nutritional supplementation. These patients could be classified as having ‘refractory anemia,’ which may be related to AI (anemia of inflammation) [5]. Patients with refractory anemia have higher mortality or transplant requirements and need new therapeutic approaches, such as bridging therapy. In a study of liver cirrhosis patients with cytopenia, 40% were found to be anemic and displayed erythroid hyperplasia in association with morphological signs of dyserythropoiesis, such as the intererythroblastic cytoplasmic bridges, normoblast bi-nuclearity, etc. [6]. It has been observed that inflammatory signals that alter BM steady-state erythropoiesis support the stress erythropoiesis or dyserythropoiesis generating erythrocytes until the inflammation is neutralized.

Understanding the pathophysiological mechanism behind the dysregulation of erythropoiesis by chronic inflammation may unmask the significant reason for anemia in patients with cirrhosis which would help form precise interventional approaches to treatment.

The present study attempted to understand the cellular and functional changes in erythroid progenitors and precursors in patients with anemia in cirrhosis. We also tried to understand erythroid cells’ differentiation and maturation defects that lead to erythropoiesis dysregulation.

## 2. Materials and Methods

Study participants: The study was conducted at the Institute of Liver & Biliary Sciences (ILBS), New Delhi, between December 2020 and April 2022, following approval from the Institutional Ethics Committee (IEC) at the ILBS (Ref: IES/2018/64NA03) on 19 December 2018. Informed consent was obtained from all participants prior to sample collection. All procedures were performed in accordance with the ethical standards of the IEC and the principles outlined in the Declaration of Helsinki (1975, as revised in 2013).

### 2.1. Inclusion Criteria

Group 1: CLD-Alcoholic (*n* = 30)—Patients with alcohol-related cirrhosis who had abstained from alcohol for no more than 3 months prior to sampling. Hemoglobin levels were <12 g/dL in females and <13 g/dL in males.

Group 2: CLD-NASH (*n* = 30)—Patients with cirrhosis due to non-alcoholic steatohepatitis. Hemoglobin levels were <12 g/dL in females and <13 g/dL in males.

Group 3: NCPF (*n* = 7)—Patients with non-cirrhotic portal fibrosis (serving as disease controls) and no evidence of advanced liver disease. Hemoglobin levels were <12 g/dL in females and <13 g/dL in males.

Group 4: Control (*n* = 3)—Patients evaluated for idiopathic thrombocytopenic purpura (ITP) or pyrexia of unknown origin (PUO) whose bone marrow evaluations were reported as normal, with no liver disease or hematologic malignancy. Hemoglobin levels were ≥12 g/dL in females and ≥13 g/dL in males.

Exclusion criteria: Patient with <18 years and >75 years of age, with any nutritional deficiency, anemia due to iron deficiency, autoimmune hemolytic anemia, aplastic anemia, myelodysplasia, hypothyroidism, hepatocellular carcinoma, co-existent with HIV infection, chronic pancreatitis, autoimmune disease, Wilson disease, any viral illness, post liver transplant patient, uncontrolled sepsis, ongoing active bleeding, any primary BM pathology, and whom refused to give consent were excluded.

BM examination: The BM aspiration was sent to a laboratory for clinical investigations. The remaining sample portion was collected by the ILBS biobank facility for research purposes.

Blood Profile: Complete blood count report (CBC), BM aspirate, iron profile, vitamin B12, thyroid profile, LDH, folate, and LFT reports were examined and recorded.

Severity scores: Liver disease severity score was measured using the Na-MELD score (Model for End-stage Liver Disease Sodium) for that total bilirubin (m/dL), INR, creatinine (mg/dL), and sodium (mm/Eq) parameters were recorded, as shown in Table 1.

### 2.2. Flow Cytometry

To examine the erythroids’ differentiation pattern, Immunostaining was performed using a whole BM aspirate sample with 1 × 10^6^ cells. RBCs were lysed using an RBC lysis solution, and cells were washed with 1X PBS. Cells were incubated with antibodies labeled with different fluorochromes at the indicated dilutions: CD11b-FITC (1:50; Clone ICRF44, BD Pharmingen, San Jose, CA, USA), CD19-FITC (1:50; Clone HIB19 [RUO], BD Pharmingen, San Jose, CA, USA), CD3-FITC (1:20; Clone HIT3a [RUO], BD Pharmingen, San Jose, CA, USA), CD71-APC (1:50; Clone M-A712 [RUO], BD Pharmingen, San Jose, CA, USA), and CD235a-PE-Cy7 (1:10; Clone GA-R2 [HIR2] [RUO], BD Pharmingen, San Jose, CA, USA). The incubation was performed at 4 °C for 15 min. After washing, 1 × 10^5^ cells were acquired using a BD FACS machine.

### 2.3. Cytokine Bead Array

Cytokine Bead Array was performed using Luminex Discovery Assay kit (Catalog no. LXSAHM-23, (HUMAN Luminex^®^ Discovery Assay, R & D SYSTEMS, Minneapolis, MN, USA) based on the Luminex xMAP Technology for the following cytokine/growth factors: CCL2, Ferritin, Flt-3, G-CSF, IFN-γ, IL-1alpha, IL-1beta, IL-1RA, IL-3, IL-5, IL-4, IL-6, IL-10, IL-12, S100A9, SCF/c-kit, TGF-alpha, TPO, TNF-alpha, TRAIL R2, and LIF.

### 2.4. Enzyme Linked Immunosorbant Assay (ELISA)

ELISA for Hepcidin (E-EL-H5497, Elabscience), TGF-β (BMS249-4, Thermofisher scientific, Waltham, MA, USA), EPO (E-EL-H3640, Elabscience, Houston, TX, USA), and Transferrin (MBS723160, Mybiosource.com) was performed using BM plasma of cirrhosis, NCPF, and control cases.

### 2.5. Mass Spectroscopy

A 50 ug protein was isolated from FACS-sorted CD71+ erythroid cells from BMMCs using a guanidinium chloride solution. In brief, proteins were isolated from the FACS-sorted CD71+ erythroid cells of the study groups. The isolated proteins were reduced, alkylated, and digested using trypsin, followed by mass spectrometry analysis. The MS/MS data were acquired and analyzed by Proteome Discoverer (version 2.0, Thermo Fisher Scientific, Waltham, MA, USA) using the human sequence (UniprotSwP_20170609, with sequences 467231 and MG_BG_UPSP with sequences 2019194).

### 2.6. Quantitative Real-Time PCR

Total cellular RNA was isolated using TRIzol reagent (15596018; Thermo Scientific, Waltham, MA, USA) and reverse-transcribed into complementary DNA (cDNA) using the GoScript^TM^ Reverse Transcription System (A5003; Promega, Madison, WI, USA) according to the manufacturer’s instructions. The cDNA was then amplified using the GoTaq^®^ qPCR Master Mix (A6001; Promega, Madison, WI, USA) for 40 cycles on a 96-well real-time PCR machine (Bio-Rad, Hercules, CA, USA) following the manufacturer’s protocol. Transcripts were amplified using gene-specific primers (Supplementary Table S9) and normalized to 18S rRNA expression.

### 2.7. Detection of Reactive Oxygen Species (ROS) by Dichloro-Dihydro-Fluorescein Diacetate (DCFH-DA)

RBC lysed 2–3 × 10^5^ BMMCs were treated with 20 μL of DCFH-DA (D6883-250MG; 149 Sigma-Aldrich, St. Louis, MO, USA) solution and incubated for 15 min at 37 °C (RT) in the dark. After washing with 1X PBS, the supernatant was discarded, and cells were resuspended in 200 μL of 1X PBS. The samples were then acquired using a BD flow cytometer.

### 2.8. Heme Quantification

A total of 1 × 10^5^ BMMCs were lysed using 200 μL of RIPA buffer then centrifuged at 13,000 rpm. at 4 °C for 15 min. The supernatant was collected in a separate tube and heme levels were quantified by measuring absorbance at 400 nm.

### 2.9. Colony-Forming Unit (CFU) Assay

To assess the colony-forming capacity of erythroid cells, a total of 2 × 10^5^ BMMCs were plated in each well with a 3.5 cm^2^ growth area. Cells were cultured in MethoCult^TM^ media (04435; 161 StemCell Technology, Cambridge, MA, USA) at 37 °C in 5% CO_2_ with ≥95% humidity for 14–16 days in an incubator. After 14 days, colonies were examined and classified based on their morphology. Each assay was performed in triplicate.

### 2.10. Culturing of Erythroid Cells

PBMCs were isolated using the ficol (LS001; Himedia, Kennett Square, PA, USA) density method and were seeded at 1 × 10^5^/mL concentration for 5–6 days in phase I erythroid proliferation medium containing StemPro^TM^ Basal Medium (09605; Stemcell Technology, Cambridge, MA, USA), 1X Pen/Strep (2211109; Thermo Fisher Scientific, Waltham, MA, USA), 2 mM L-Glutamine (25030081; Thermo Fisher Scientific, Waltham, MA, USA) 1X Nutrient Supplement, 100 ng/mL rhSCF (11343325; ImmunoTools, Friesoythe, Germany) 20 ng/mL rhIL-3 (203-IL-010; R&D System, Minneapolis, MN, USA), 100 ng/mL rhFLT-3 ligand (11343305; ImmunoTools, Friesoythe, Germany), 20 ng/mL rhIL-6 (206-IL-010; R&D System, Minneapolis, MN, USA) at 37 °C in 5% CO_2_ to promote cell expansion. After this initial phase, cells were transferred to phase II erythroid differentiation medium containing StemPro^TM^ Basal Medium, 1X Pen/Strep, 2 mM L-Glutamine, 1X Nutrient Supplement, 20 ng/mL rhSCF, 2 ng/mL rhIL-3, 900 ng/mL ferrous sulphate (F7002; Sigma-Aldrich, St. Louis, MO, USA), 90 ng/mL Ferric nitrate (F3002; Sigma, Kanagawa, Japan), 1.8 uL β-mercaptoethanol (2176575; Thermo Fisher Scientific, Waltham, MA, USA), and 2 U/mL EPO (286-EP-250, R&D System, Minneapolis, MN, USA) for an additional 6–8 days to promote erythroid differentiation.) for a further 6–8 days.

### 2.11. Statistical Analysis

The results are expressed as the mean ± SD unless indicated otherwise. Statistical significance between two groups was determined using an unpaired two-tailed Student’s *t*-test or Welch’s *t*-test, as appropriate. All analyses were performed using GraphPad Prism Software (version 6; GraphPad). All correlations were performed using Spearman correlation analysis, and R^2^ > 0.5, *p* < 0.05 were considered statistically significant.

## 3. Results

### 3.1. Baseline Characteristics

The study included three major groups, alcoholic and nonalcoholic liver disease (*n* = 30 each), non-cirrhotic portal hypertension (NCPF, *n* = 7), and control (*n* = 3). The baseline demographic characteristics were recorded at the time of enrollment in the study. Enrolled subjects had a mean age of 50.97 ± 11.08, including males and females (51 Males: 9 Females) The mean MELD-Na score of these CLD patients was 19.47 ± 7.39. All subjects included had no nutritional deficiency, as shown in Table 1. All CLD patients were anemic, with hb levels < 12 g/dL in females and <13 g/dL in males. NCPF patients were considered as disease control and control group were those suspected of idiopathic thrombocytopenic purpura (IPA) or the patient who came for PUO diagnosis, but the BM report came out normal.

### 3.2. Study of Erythroid Population and Association with Anemia in Cirrhosis

**Figure 1:**
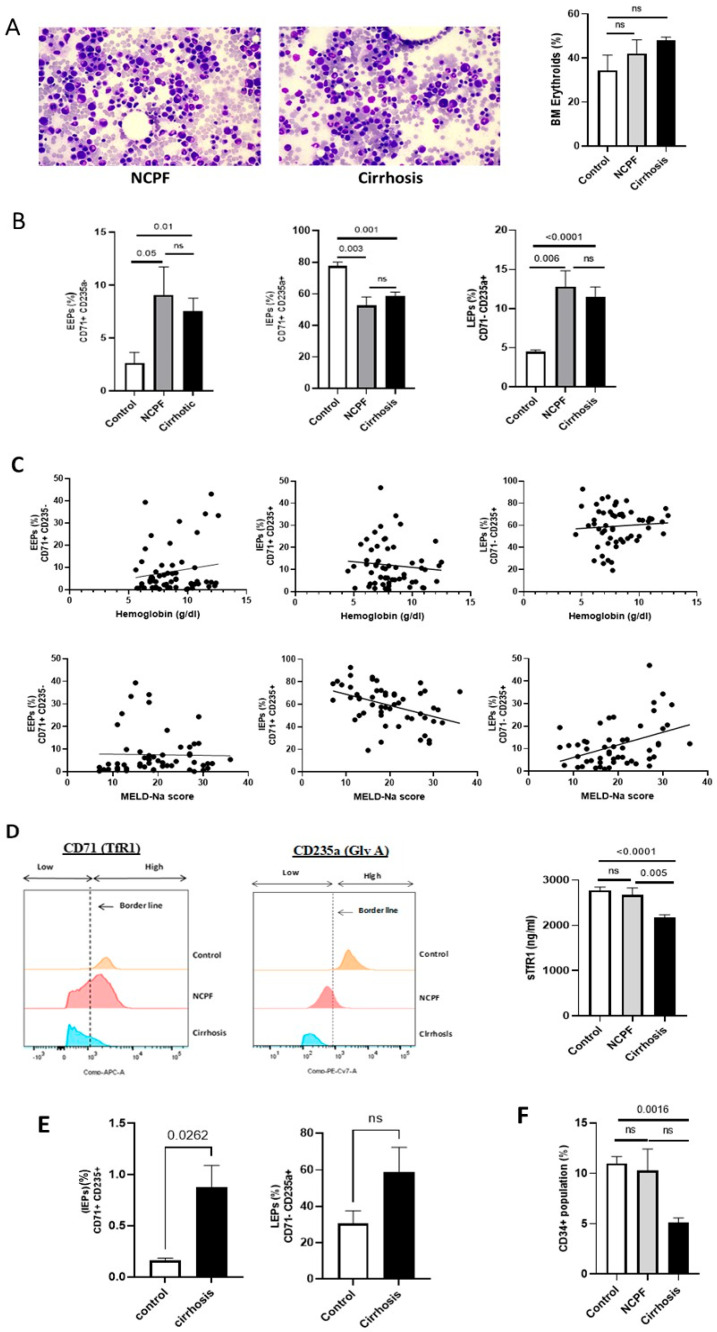
Comparative analysis of BM characteristics in cirrhosis. (**A**) Geimsa stained BM slide of NCPF and cirrhosis (left) showing erythroid hyperplasia (encircled in red) and statistical graph representing erythroid cells (%) (right) in BM of cirrhosis (*n* = 60), NCPF (*n* = 7) and control (*n* = 3). (**B**) Erythroid cell population (%) EEPs, IEPs, and LEPs were analyzed in cirrhosis (*n* = 60), NCPF (*n* = 7), and control (*n* = 3). (**C**) Linear regression graphs representing spearman correlation of hemoglobin (g/dl) and MELD-Na scores with different erythroid populations: EEPs, IEPs, and LEPs. (**D**) FACS based mean fluorescence intensity (MFI) graph (left) of CD71+ and CD235a+ on erythroid cells were analyzed. On the basis of the control border line (dash line) was drawn, left side of line shows population with low marker intensity and right side population have high intensity of markers. ELISA of soluble TfR1 (sTfR1) (right) was analyzed in cirrhosis (*n* = 60), NCPF (*n* = 7), and control (*n* = 3). (**E**) Erythroid progenitor (%) population: IEPs and LEPs were analyzed in cirrhosis (*n* = 5) and control (*n* = 3). (**F**) Total CD34+ HSC population (%) was analyzed in cirrhosis (*n* = 35), NCPF (*n* = 5) and control (*n* = 3). Exact *p*-values are shown for significant comparisons, while ‘ns’ indicates non-significant differences.

The microscopic examination of the BM smear slide showed erythroid hyperplasia (Figure 1A) in patients with cirrhosis and NCPF as compared to the control. Similar results were found when compared to a different etiology (Supplementary Figure S1A). Flow cytometry results showed that EEPs were significantly (*p* < 0.05) increased in both cirrhosis (8.33 ± 10.7%) and NCPF (9.07 ± 6.99%) as compared to the control (2.6 ± 1.8%). Conversely, IEPs were significantly (*p* < 0.05) decreased in cirrhosis (58.5 ± 17.16%) and NCPF (52.68 ± 14.15%) as compared to control (77.6 ± 4.32%). LEPs were significantly (*p* < 0.05) increased in cirrhosis (11.5 ± 9.5%) and NCPF (12.77 ± 5.45%) as compared to control (4.48 ± 0.4%) (Figure 1B, Table 2) (Supplementary Figure S1B–D).

In turn, IEPs were inversely correlated with the patient’s hemoglobin levels (r = −0.691) and Na-MELD score (r = −0.442), whereas LEPs were positively correlated for the same (Hb, r = 0.807; Na-MELD score, r = 0.387 (Figure 1C) (Supplementary Figure S1E,F; Supplementary Table S1). Expression of transferrin receptors on erythroid progenitors caused changes in a disease’s condition. We observed that the maximum population of the cirrhosis group had low expression of transferrin receptor, further validated by quantitating soluble transferrin receptor 1 (sTfr1) in BM plasma and found a significant (*p* < 0.05) reduction in cirrhosis (2093 ± 580 ng/mL) as compared to NCPF (2677 ± 428 ng/mL) and control (2772 ± 172 ng/mL) (Figure 1D) (Supplementary Figure S1G). Moreover, we found an increase in IEPs (0.88 ± 0.46%) and LEPs (59 ± 29.5%) in the peripheral blood of patients with cirrhosis as compared to control [IEPs (0.16 ± 0.03%) and LEPs (30.5 ± 12.2%)]. IEPs were found to be significantly (*p* < 0.05) high in patients with cirrhosis (Figure 1E, Table 2). BM-isolated CD34^+^ hematopoietic stem cells (HSCs) were significantly decreased in cirrhosis (6.56 ± 3.61%) compared with controls (9.16 ± 4.11%; *p* < 0.05). However, when compared with the NCPF group (9.8 ± 4.85%), this difference was not statistically significant. (Figure 1F, Table 2) (Supplementary Figure S1H,I). Decreased TFR in BM erythroid cells and immature erythroid cells in patient’s peripheral blood indicate dysregulation in proliferation and maturation process. Correlation analysis of erythroid progenitors with patient age is given in Supplementary Table S12.

### 3.3. Intracellular Alteration Ceases the Growth and Maturation of Erythroid Cells

**Figure 2:**
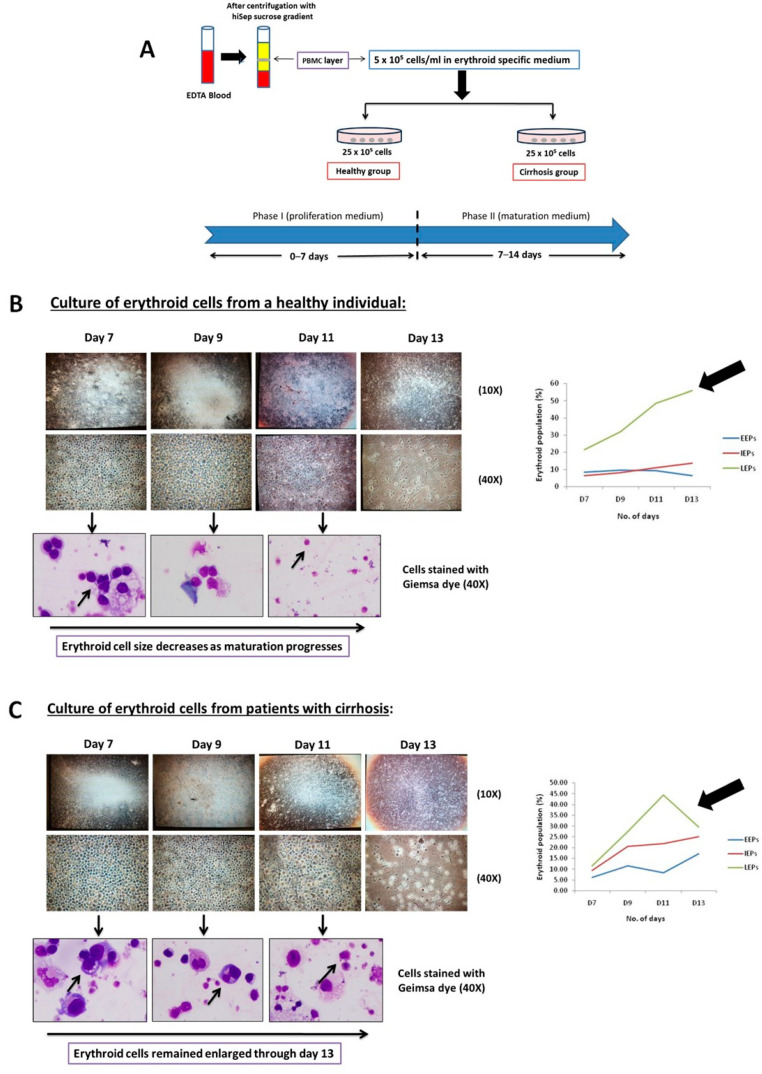

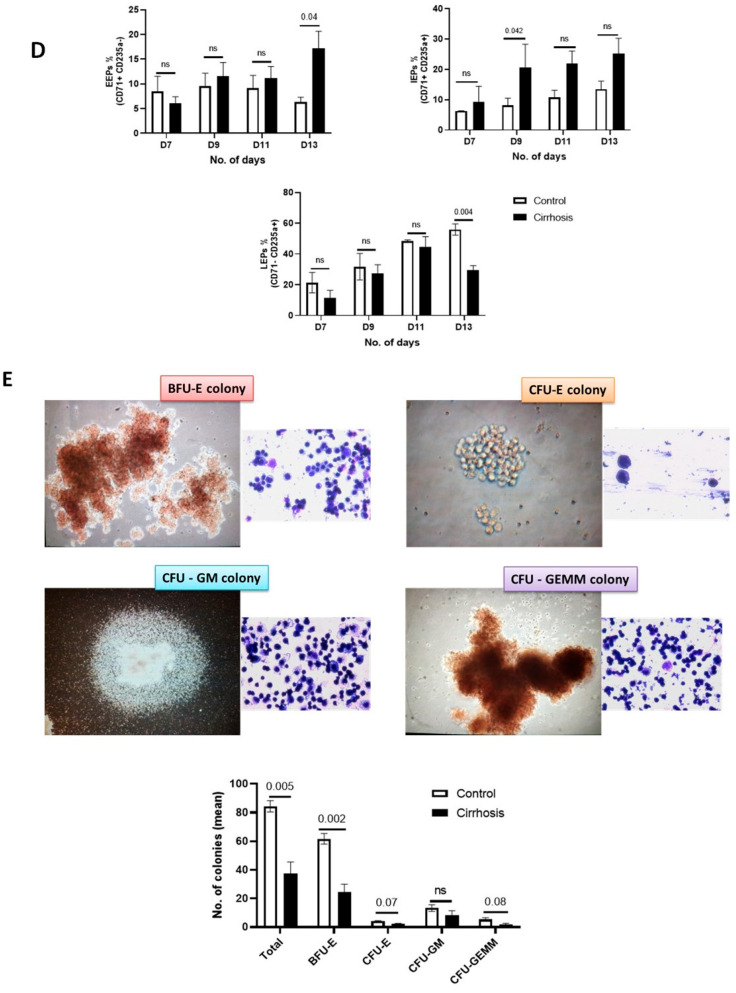
Exploring bone marrow erythroid cells and in vitro culture outcomes. (**A**) Flow chart of experimental design. (**B**,**C**) Representative images of erythroid cultured cells from (**B**) healthy individuals (n = 3) and (**C**) patients with cirrhosis (n = 3). Images were captured using an inverted microscope at 10× and 40× magnification. Cells were stained with Giemsa dye, and additional images were taken at 40× magnification using a compound microscope. Line graph representing FACS results showing the count (%) of the erythroid population: EEPs, IEPs, and LEPs from day 7 to day 13 of culture. (**D**) Statistical graphical representation of FACS results of previously cultured erythroid population (%): EEPs, IEPs, and LEPs from day 7 to day 13. (**E**) Representative images of in vitro colony-forming unit (CFU) assays taken at 40× magnification, showing four different colonies: BFU-E (early erythroid), CFU-E (mature erythroid), CFU-GM (granulocyte-macrophage), and CFU-GEMM (granulocyte, erythroid, macrophage, and megakaryocyte). Colonies were formed after 14 days of PBMC culture using MethoCult media. Statistical graphical representation of four different colonies after day 14 in cirrhosis and compared with control BM. Exact *p*-values are shown for significant comparisons, while ‘ns’ indicates non-significant differences.

Next, we accessed the erythropoiesis process of the erythroid cells taken from a patient with cirrhosis in vitro provided normal conditions until day 14, analysis was performed through microscopy and flow cytometry (Figure 2A). We found increased EEPs and IEPs with distinct morphological characteristics with an increased nuclear/cytoplasmic ratio as compared to the erythroid cells from the control group (Figure 2B,C). IEPs were found to be increased from day 7 until day 13, but significantly increased (*p* < 0.05) on day 9 in cirrhosis compared to the control (Figure 2D). This suggests that the growth of erythroid cells is inhibited at the EEP stage, preventing further differentiation. This indicates dysregulated erythropoiesis, even when cultured in a standard environment with enriched culture conditions.

To further investigate the functionality of cirrhotic erythroid cells, we performed the colony-forming unit (CFU) assay. The results showed that the total number of colonies formed significantly decreased in cirrhosis compared to the control, as shown in Supplementary Table S2. BFU-E colonies were particularly significantly reduced (*p* < 0.05) as compared to other CFU-E, CFU-GM, and CFU-GEMM in cirrhosis as compared to controls (Figure 2E), suggesting that the proliferation and differentiation capacity of erythroid colonies are significantly reduced in cirrhosis.

### 3.4. Proteomic Evaluation of CD71+ Erythroid Population in Cirrhosis and Its Association to Anemia

**Figure 3:**
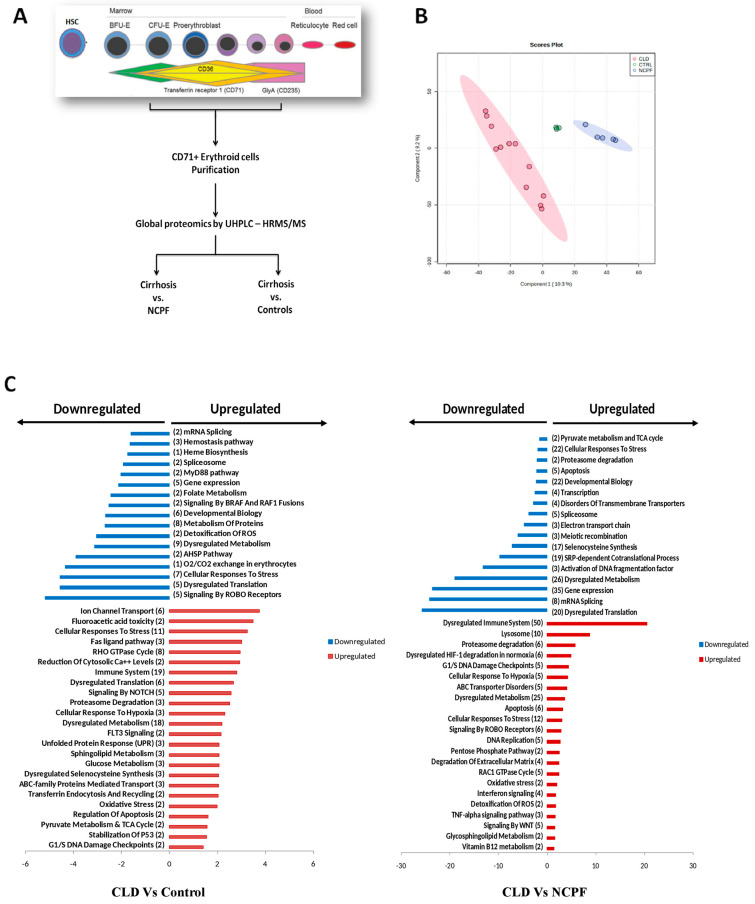
Proteomic profiling of CD71+ erythroid cells. (**A**) Flow chart of proteomics experiment of CD71+ erythroid population. (**B**) LS-DA plot shows three distinct populations including cirrhosis (*n* = 12) [pink], NCPF (*n* = 5) [blue], and control (*n* = 3) [green]. (**C**) Upregulated (red) and downregulated (blue) pathways drawn from differentially expressed proteins (DEPs: cirrhosis (*n* = 12) versus control (*n* = 3) (left) and cirrhosis (*n* = 12) versus NCPF (*n* = 5).

So, to map their molecular profile, CD71+ erythroid cells were purified from the BM of cirrhosis, NCPF, and control subjects (Figure 3A). When compared to control, proteomic analysis of purified CD71+ erythroid cells from patients with cirrhosis were identified with 120 differentially expressed proteins (DEPs), of which 93 were up-, and 26 were down-regulated (Supplementary Table S3). In comparison to NCPF, erythroid cells from cirrhosis showed a total of 196 proteins as DEPs, out of which 114 up-, and 80 were down-regulated [fold change [FC] > 1.5, (*p* < 0.05)], (Supplementary Figure S2A; Supplementary Table S4). Partial least squares discriminant analysis (PLS-DA) (Figure 3B) and clustering analysis distinctly segregated cirrhosis erythroid population from NCPF or controls (Supplementary Figure S2B). Interestingly, erythroid cells from patients with cirrhosis showed significantly upregulated pathways linked to the cellular response to stress, dysregulated translation, transferrin endocytosis and recycling, oxidative stress, dysregulated metabolism, Fas ligand pathway, fluoroacitic acid toxicity, lysosome, dysregulated HIF-1 pathway, pentose phosphate pathway, degradation of extracellular matrix, and G1/S DNA checkpoints. At the same time, pathways linked to heme biosynthesis, folate metabolism, mRNA splicing, detoxification of ROS, O_2_/CO_2_ exchange in erythrocytes, apoptosis, pyruvate metabolism, TCA cycle, spliceosome, and SRP-dependent Co-translational processes were significantly (*p* < 0.05) downregulated (Figure 3C). The mean decrease in accuracy was also observed (Supplementary Figure S2C). Together results show that a cirrhotic environment may cause changes in erythroid cells.

We also studied the pathway alteration in cirrhosis with two different etiologies: alcohol related-cirrhosis and NASH cirrhosis and found significant differences between two groups (Supplementary Figure S2D,E). Proteomic analysis of the CD71+ erythroid population of cirrhosis led to the identification of a total of 137 differentially expressed proteins (DEPs), of which a total of 60 were up- and 76 were down-regulated when compared to controls (Supplementary Figure S2F,G; Supplementary Table S5). Erythroid population proteomics data were also correlated with different Na-MELD scores. We found a total of 111 proteins showed direct correlation whereas a total of 120 proteins were inversely correlated to the severity of liver cirrhosis (Supplementary Figure S3A,B; Supplementary Table S6).

### 3.5. Pathway Activity Analysis of CD71+ Erythroid Population and Their Association with Anemia

**Figure 4:**
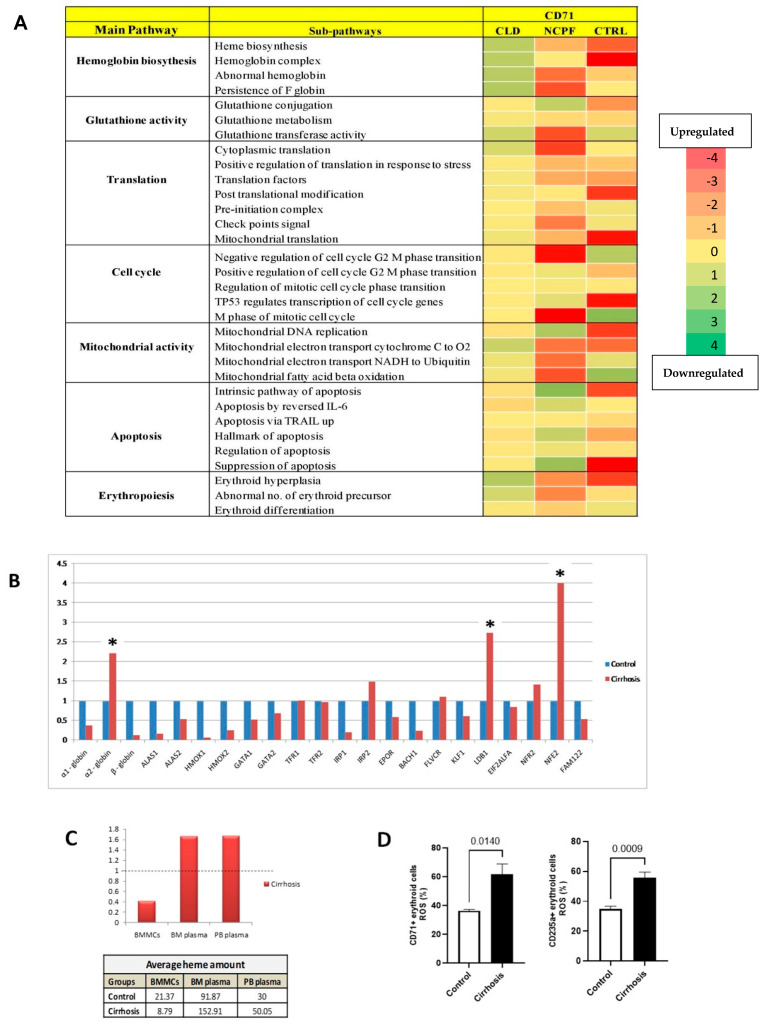
Hematological analysis of pathway activity, gene expression, heme levels, and ROS in cirrhosis. (**A**) Pathway activity analysis of CD71+ erythroid population in cirrhosis compared to NCPF and control. (**B**) Quantitative PCR results of genes related to hemoglobin synthesis and erythropoiesis (*n* = 12). * Asterisks indicate a two-fold increase in mRNA levels. (**C**) Measurement of heme in average (table) and in fold change in BMMCs, BM plasma, and PB plasma in cirrhosis as compared to control (graph). The cut-off for control is represented by a dashed line (--). (**D**) Measurement of ROS (reactive oxygen species) in BMMCs through FACS in cirrhosis (*n* = 7) as compared to control (*n* = 3). Exact *p*-values are shown for significant comparisons.

We next investigated the activity levels of key erythroid-related pathways, such as heme biosynthesis, glutathione metabolism, translation, cell cycle, mitochondrial activity, apoptosis, and erythropoiesis, among the three study groups (Control, NCPF, and CLD) (Figure 4A) (Supplementary Figure S3C). Mean ± SD values and detailed statistical comparisons are provided in Supplementary Table S7. The activity of heme biosynthesis was significantly reduced in the CLD group compared to both control and NCPF (*p* = 0.0481 and *p* = 0.0240, respectively), suggesting impaired heme production in advanced liver disease. Apoptotic pathway activity was significantly lower in NCPF in comparison to both control and CLD (*p* = 0.0103 and *p* = 0.0115, respectively), whereas CLD showed a modest increase compared to NCPF but had no significant difference from control (*p* = 0.1170). Translation pathway activity was significantly lower in CLD compared to NCPF (*p* = 0.0003), indicating reduced protein synthesis at advanced disease stages. Erythropoiesis was also significantly decreased in CLD compared to NCPF (*p* = 0.0158), whereas glutathione activity, mitochondrial activity, and cell cycle pathways did not reveal statistically significant differences between the groups. Overall, these findings highlight distinct alterations in specific erythroid pathways, with the greatest level of suppression observed in the CLD group.

We aimed to investigate erythropoiesis at the gene level to validate our proteomics findings. The gene expression analysis of the erythroid cells from patients with cirrhosis revealed significant dysregulation of genes involved in erythropoiesis and heme synthesis (Control, *n* = 3; Cirrhosis, *n* = 10) (Figure 4B), (Supplementary Tables S8 and S9). Expression of genes responsible for hb syntheses such as α1-globin and β-globin were downregulated, whereas α2-globin was upregulated, suggesting impaired hb synthesis in cirrhosis. This is consistent with the decreased levels of ALAS1 and ALAS2, enzymes involved in heme synthesis. HMOX-1 and HMOX-2 gene expression were downregulated, indicating impaired heme metabolism in cirrhosis. The downregulation of GATA-1, along with the unchanged expression of GATA-2 in cirrhosis, suggests a partial impairment of their regulatory functions in erythropoiesis. The erythropoietin receptor was also found to be downregulated. The expression of iron metabolism-related genes, such as TfR1, TfR2, IRP1, and IRP2, were also studied in cirrhosis. This divergent expression pattern, with suppression of TfR1 and induction of TfR2, suggests altered iron uptake and regulatory mechanisms during disordered erythropoiesis in cirrhosis. Iron regulatory protein (IRP2) was found to increase, whereas IRP1 was decreased, suggesting dysregulated iron regulatory pathways in cirrhosis. The dysregulation of several transcription factors, such as BACH1, NFR2, LDB1, NFE2, and KLF1, may also contribute to defective erythropoiesis in cirrhosis. Results showed that low BACH1 and high NFR2 expression make the erythroid cells more susceptible to heme toxicity. FLVCR, which protects developing erythroid cells from heme toxicity, showed no appreciable difference compared with control samples.

LDB1 and NFE2 are involved in terminal erythroid differentiation, were increased expression. EIF2α is known to be activated under low-heme conditions, and although our dataset did not reveal appreciable differences, this pathway may still play a role in stress-associated regulation of erythropoiesis. FAM122A is a housekeeping gene whose downregulation suggests its role in inhibiting erythroid differentiation.

Heme is necessary for hemoglobin molecules; therefore, we quantified the heme in BMMCs, BM plasma, and PB plasma of the same cirrhotic patients. We observed reduced cellular heme levels in cirrhosis compared to controls, while higher levels of free heme were found in bone marrow (BM) plasma and peripheral blood (PB) plasma, which could be due to hemolysis (Figure 4C). We also observed a significant (*p* < 0.05) increase in ROS levels in both the CD71+ early and CD235a+ late erythroid population (Figure 4D), which supports the notion that erythroid cells are susceptible to oxidative stress and free radicals.

### 3.6. Treatment with Cirrhotic Plasma Causes the Maturational Arrest of IEPs

**Figure 5:**
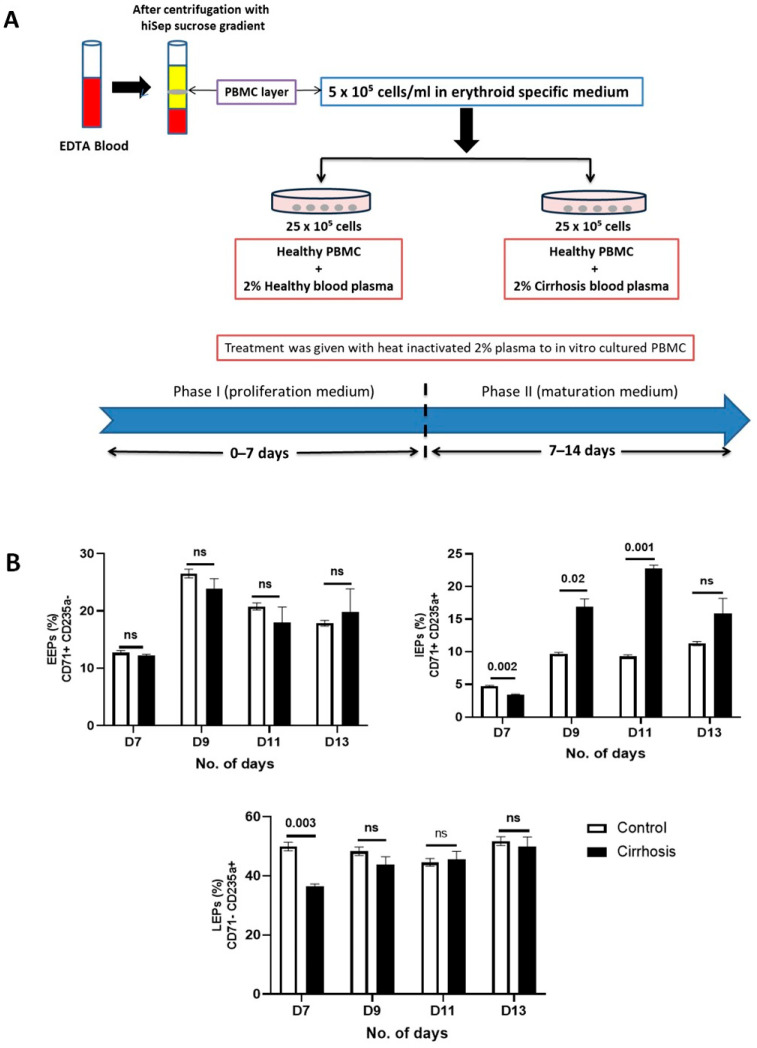
In vitro erythroid cell population analysis with plasma treatment. (**A**) Experimental design for in vitro erythroid cell culture with plasma treatment. (**B**) FACS-based data showing different population (%) EEPs, IEPs, and LEPs at day 7, day 9, day 11, and day 13 in culture: healthy PBMC with 2% healthy plasma treatment (*n* = 3) and healthy PBMC with 2% cirrhotic plasma treatment. Exact *p*-values are shown for significant comparisons, while ‘ns’ indicates non-significant differences.

Until now, we have observed issues in erythropoiesis in patients with cirrhosis and the BM milieu containing various altered erythropoiesis regulating factors and inflammatory cytokines. Further, to elucidate this, we performed in vitro cultures of erythroid progenitor cells that were expanded from PBMCs obtained from a healthy person and treated with the peripheral blood plasma of a cirrhotic patient. This can help simulate the conditions in cirrhotic BM and evaluate the impact of the toxins and cytokines on the erythroid cells (Figure 5A). On day 9 and 11, only IEPs were significantly increased (*p* < 0.05) in the treated group. Finally, on day 13, IEPs were raised but EEPs and LEPs did not show any difference from day 9 until day 11 in the treated group as compared to the control (Figure 5B). The results showed that incubation with patient plasma significantly decreased the IEPs and LEPs on day seven but did not affect EEPs suggesting that plasma from cirrhosis patients severely affects the differentiation and proliferation efficiency of erythroid cells.

### 3.7. Association of Altered BM Milieu with Anemia

**Figure 6:**
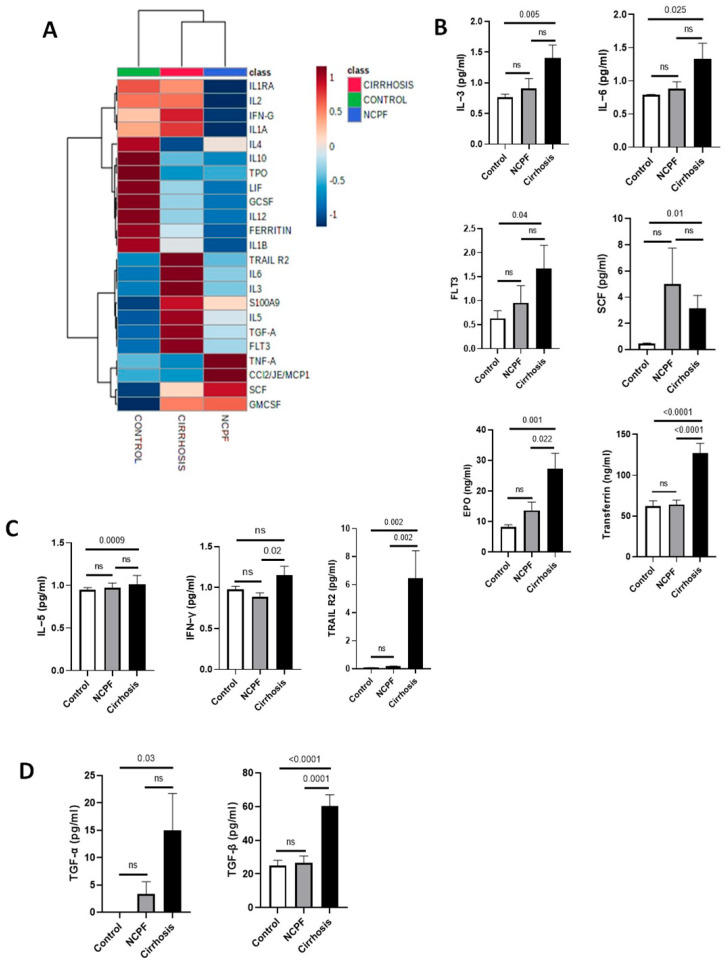
Heatmap analysis of erythropoiesis regulating factors, cytokines, and growth factors. (**A**) Clustered heatmap was generated using metaboanalyst 5.0. Comparison of three different groups, cirrhosis (*n* = 60), NCPF (*n* = 7), and control (*n* = 3) was performed against BM’s 23 erythropoiesis regulating factors/cytokines/growth factors. Expression of (**B**) erythropoiesis regulating agents; IL-3, IL-6, FLT3, SCF, EPO, and transferrin, (**C**) pro–inflammatory cytokines: IL-5, IFN-γ, and TRAIL R2, (**D**) anti–inflammatory cytokines: TGF-α and TGF-β in cirrhosis as compared to NCPF and control. Exact *p*-values are shown for significant comparisons, while ‘ns’ indicates non-significant differences.

To understand potential factors influencing or modulating normal erythropoiesis, we analyzed the panel of 26 erythropoiesis regulating factors/cytokines/growth factors by cytokine array (Figure 6A) and ELISA using BM plasma of patient and control groups (Supplementary Table S10). Erythropoiesis-regulating factors such as IL-3, IL-6, FLT3, and SCF were significantly (*p* < 0.05) increased in cirrhosis compared to the control, but no significant difference was observed when compared with NCPF (Figure 6B). In contrast, both EPO and transferrin levels were significantly increased in cirrhosis compared to both control and NCPF. No significant difference in TPO, hepcidin, and ferritin levels were found (Supplementary Figure S4A). We found a significant (*p* < 0.05) increase in IL-5 and TRAIL R2 levels in cirrhosis compared to the control, whereas TRAIL R2 (but not IL-5) was significantly increased when compared to NCPF. Whereas, IFN-γ levels were significantly higher in the cirrhosis group compared to NCPF (*p* = 0.02), while no significant difference was observed when compared to the control group (Figure 6C). IL-1α, IL-1β, IL-2, TNF-α, S100-A9, and CCL2/JE/MCP-1 did not show significant differences, whereas IL-12 showed an important (*p* < 0.05) reduction as compared to the control (Supplementary Figure S4B). We found a significant (*p* < 0.05) decrease in pro-inflammatory cytokines such as IL-4, IL-10, G-CSF, and LIF. IL-1Ra levels were markedly reduced in the NCPF group compared to the control group (*p* < 0.05) and significantly elevated in the cirrhosis group relative to the NCPF group (*p* < 0.05); however, there was no significant difference (*p* > 0.05) between the cirrhosis and control groups (Supplementary Figure S4C). TGF-α was significantly increased in cirrhosis compared to controls, while no significant difference was observed between cirrhosis and NCPF. TGF-β was significantly increased in cirrhosis compared to both NCPF and controls (*p* < 0.05), indicating erythropoiesis dysregulation and induction of inflammation (Figure 6D). Correlation analysis was performed of all cytokines with hemoglobin of patients with cirrhosis (Supplementary Table S11) and found that IL-6 and G-CSF were negatively correlated with hemoglobin. We have also found a positive correlation between IL-3 and ROS generated by erythroid cells (Supplementary Table S12). Altogether, we observed dysregulated erythropoiesis in patients with cirrhosis that could be due to abnormal levels of erythropoiesis-regulating agents and increased levels of inflammatory cytokines that together cause stress on developing erythroid cells, potentially interference with their maturation and function have been observed.

## 4. Discussion

Anemia in a patient with cirrhosis is an important, clinically pertinent, but often neglected disease association. Relevant guidelines emphasize an algorithmic approach to treating cirrhotic patients with acute variceal bleeding, but daily management in hospital and outpatient settings pose several dilemmas such as whether anemia is a disease complication or part of the disease spectrum? Should iron, folic acid, and vitamin B complex supplementation and nutritional advice be sufficient in treating patients having persistently low hemoglobin?

Therefore, understanding the underlying pathophysiological processes leading to anemia, its diagnosis, and management are important aspects of treatment. However, since this is an observational study, our findings demonstrate associations rather than establishing direct causality. Despite having no significant change in hemocrit and MCV values, patients still develop anemia due to unknown pathological reasons. The absence of stainable iron in the marrow does not necessarily indicate total iron depletion, as non-stainable forms like ferritin may still be present. Chronic gastrointestinal blood loss can induce erythroid hyperplasia, leading to depletion of iron stored in the bone marrow macrophage system. It shows that the etiology underlying this persistent anemia appears to be independent of any deficiency and bleeding disorder. Therefore, the analysis of the erythroid population may offer valuable insights into the differentiation patterns observed in cirrhotic patients. Based on the results, our study found altered erythroid populations in cirrhosis and NCPF, with increased EEPs and LEPs and decreased IEPs as compared to the control, possibly due to an increase in the demand for erythrocytes in these patients as a compensatory mechanism to overcome anemia [7]. Erythroid populations also showed significant correlation with MELD-Na score and hb. We found lower expression of TfR1 on erythroid cells of cirrhosis as compared to NCPF and controls. This is consistent with previous studies that have shown that decreased TfR1 expression can lead to iron restriction in erythroid cells [8]. Elevated levels of IEPs in the peripheral blood of cirrhosis patients may indicate a compensatory response to dysregulated erythropoiesis and impaired differentiation. In our results, in vitro culture of cirrhotic cells showed an increase in EEPs and IEPs and a decrease in LEPs, even provided the normal condition as the cells were already pre-exposed to continuous inflammatory conditions which resulted in altered differentiation towards matured and functional erythrocytes, which was confirmed by reduced erythroid colony formation in the CFU assay in patients with cirrhosis.

To focus on the underlying mechanisms of such alterations of erythropoiesis, proteomics analysis was performed for the CD71+ erythroid population. Oxidative stress, dysregulated translation, and downregulated metabolism can activate the UPR pathway or lead to apoptotic death in erythroblasts of cirrhosis. We also found dysregulated metabolism and an increase in pentose phosphate pathways. In addition, we found downregulated pathways related to the cellular stress response. Cellular stress response activation helps the tissue adapt to the damage and restore function. If this repair or adaptation fails or the stress is too severe and persistent, the cell will eventually die and might release DAMPs [9,10]. A reduced or defective ROS detoxification pathway causes excessive ROS and oxidative damage to the cell membrane. Increased deformability and splenic sequestration of RBCs result in a reduced life span of RBCs leading to anemia [11]. The significant decline in heme biosynthesis and erythropoiesis observed in the CLD group probably indicates impaired red blood cell production in advanced liver disease. Interestingly, apoptotic activity was reduced in NCPF when compared to both control and CLD, indicating that the early phases of the disease may involve suppression of programmed cell death, which subsequently increases in advanced cirrhosis.

The heme component is required to synthesize a complete hemoglobin molecule; defects in heme synthesis during erythroid lineage result in genetic disorders like sideroblastic anemias or erythropoietic porphyrias [12]. We found genes involved in hemoglobin synthesis such as α1-globin, β-globin, ALAS1, ALAS2, HMOX1, HMOX2, IRP1, and BACH1 were downregulated whereas α2-globin, IRP2 were upregulated and had normal TfR1 and TfR2 levels. Decreased expression of ALAS2 enzyme in erythroblasts impairs heme biosynthesis and reduces PPIX production to use all the imported iron. Mice with induced stress conditions have shown HMOX-1 haploinsufficiency results in disrupted erythroblast differentiation [13]. In β-thalassemia patients, excess free α-globin accumulates in the erythroid, leading to hemolysis and ineffective erythropoiesis [14]. Transcription factors involved in erythropoiesis such as GATA1, GATA2, EPOR, and KLF1 were downregulated whereas LDB1, NFR2, and NFE2 were upregulated. Overexpression of LDB1 and NF-E2 delays or inhibits erythroid maturation [15,16]. The GATA1 expression is significant for erythroid development, inflammatory molecules such as TNF-α, IL6, and IFNγ target the GATA1 in erythroid progenitors and promote lymphoid development [17].

Pro-inflammatory cytokines manifest suppressive effects on erythropoiesis that lead to alterations of iron homeostasis, differentiation process, and membrane of produced erythrocyte that impair its survival, causing anemia of inflammation. Here, we have shown that increased IEPs may be due to the effect of cirrhosis that creates stress in IEPs and causes maturation arrest. This can result in defective or dysregulated erythropoiesis and potentially lead to anemia, as the production of mature, functional erythrocytes may be impaired; therefore, we assessed the BM milieu to investigate the potential factors responsible for dysregulated erythropoiesis and erythroids’ maturational arrest. We found altered levels of erythropoiesis regulating agents and cytokines that act at different stages of erythropoiesis. We found an increased levels of IL-3, IL-6, FLT3, SCF, transferrin, and EPO, which promote the proliferation of erythroid progenitors and increase RBC production. Increased production of EPO prevents apoptosis of erythroid progenitors and precursors containing precipitated α-globin chains [18,19,20] leading to an increased rate of erythroid proliferation resulting in impaired erythroid maturation [21], thus establishing a vicious cycle as seen in β-thalassemia patients. Interestingly, IL-6 which regulates erythropoiesis at late stages also acts as a pro-inflammatory cytokine and increased IL-6 together with hepcidin promotes iron-restricted anemia [22]. In our study, we found a significant increase in pro-inflammatory cytokines such as IL-5, IFN-γ, and TRAIL-R2 and decrease in anti-inflammatory cytokines such as IL-Ra, IL-4, IL-10, G-CSF, and LIF, except TGF-α and TGF- β which were significantly high in patients with cirrhosis. Many studies showed that TNF-α, IFN-γ, TRAIL-R2, and IL-1 are involved in inhibiting the proliferation and differentiation of erythroid progenitor cells [23,24,25,26,27,28]. In contrast, IFN-γ, TNF-α, and TGF-β1 are potent inhibitors of erythropoiesis [29]. TRAIL, a member of the TNF-related proteins, can induce apoptosis in different committed lineages of cells [30]. TRAIL exerts its inhibitory effect on erythroid development by activating MAP kinase ERK1/2 pathway correlated with expression of TRAIL-R2, leading to the development of anemia [31].

Therefore, it is worth understanding anemia as part of the disease process rather than simply as a complication of the disease.

## 5. Conclusions

Our study shows that cirrhosis is associated with profound defects in erythropoiesis, characterized by impaired erythroid progenitor maturation (reduced BFU-E and CFU-E colonies) and altered transferrin receptor expression. These cellular alterations are exacerbated by abnormal cytokine profiles as well as the dysregulation of iron metabolism. Thus, our results support the concept that anemia in cirrhosis is not merely a secondary complication, but rather an inherent part of the disease process, resulting from multiple overlapping mechanisms such as impaired erythroid maturation, iron restriction, and inflammatory cytokine dysregulation. Understanding these mechanisms reveals new insights into the pathophysiology of cirrhosis-associated anemia and identifies potential therapeutic targets.

## 6. Limitations

This research offers significant insights into erythropoiesis in the context of liver disease; however, there are certain limitations that need to be recognized. The control group comprised three bone marrow samples from patients assessed for ITP or PUO, which, while rigorously chosen, may not represent the broader population of healthy individuals. Although strict exclusion criteria were implemented, some confounding factors, including subclinical infections and comorbidities, could have impacted erythropoiesis and should be further examined. This is an observational study. Future studies using both in vitro and in vivo models are essential for a more comprehensive understanding of the functional basis behind the observed changes.

## Figures and Tables

**Table 1:** Clinical parameters were analyzed in cirrhosis, NCPF, and control.

	(%)	Control	NCPF	Cirrhosis (CLD)	Etiology-Based Division of Patients with Cirrhosis		
					Alcoholics	NASH	*p*-Value
Patient no. (N)		3	7	60	30	30	-
Age (average)		41.33 ± 12.1	47.28 ± 14.05	50.97 ± 11.08	47.7 ± 10.33	54.23 ± 10.99	0.0211 *
Gender		Male-2, Female-1	Male-2, Female-5	Male-51, Female-9	all Male	Male-21, Female-9	-
MELD-Na (average)		-	14.4 ± 2.3	19.47 ± 7.39	22.53 ± 6.72	15.94 ± 6.58	0.0005 ***
Iron stores (%)	nil	0	71	20.7	20	27	
	grade I	33	0	31	30	30	
	grade II	33	0	17.2	20	13	
	grade III	33	29	13.8	10	17	
	grade IV	0	0	5.2	7	3	
	grade V	0	0	12.1	13	10	
RBC (3.8–4.8 × 10^9^/L)		4.5 ± 0.94	3.5 ± 0.48	2.92 ± 0.77	2.8 ± 0.68	3.03 ± 3	0.2667, ns
Hemoglobin (12–15 g/dL)		13.73 ± 2.02	9.54 ± 1.02	8.73 ± 2.02	8.56 ± 1.9	8.9 ± 2.1	0.59, ns
Hemocrit (36–46%)		40.2 ± 6.47	29.5 ± 2.7	26.62 ± 6.19	25.77 ± 5.8	27.43 ± 6.54	0.316, ns
MCV (83–101 fL)		92 ± 6.2	84.18 ± 11.3	92.26 ± 10	92.7 ± 8.85	91.79 ± 11.13	0.724, ns
RDW (11.6–14%)		13.93 ± 1.79	18.6 ± 7.5	17.56 ± 2.65	17.8 ± 2.69	17.32 ± 2.63	0.500, ns
Erythroid (%)		34.3 ± 12.09	42.5 ± 17.85	47.93 ± 12.39	46.16 ± 10.87	50.26 ± 13.79	0.271, ns
LDH (265–500 IU/L)		-	527 ± 100	490 ± 219	461 ± 192.5	533 ± 255	0.415, ns
Vitamin B12 (250–1100 pg/mL)		-	889 ± 570	1874 ± 1813	2520 ± 2115	951 ± 608	0.048 *
TSH (0.34–5.6 μIU/mL)		-	-	3.32 ± 2.46	2.14 ± 2.23	4.27 ± 2.23	0.05, ns
Ferritin (30–280 μg/L)		-	435 ± 130	286 ± 304	378 ± 361	162.7 ± 140.6	0.049 *
Serum iron (30–160 μg/dL)		-	31.5 ± 12	72.3 ± 40.58	72.8 ± 39.82	71.65 ± 43	0.934, ns
UIBC (155–355 μg/dL)		-	-	190 ± 143	-	-	
TIBC (225–425 μg/dL)		-	199 ± 146	231 ± 132	210 ± 145	260 ± 115	0.255, ns
T.saturation (20–50%)		-	37.02 ± 41.7	47.3 ± 47.7	60.74 ± 63	31.15 ± 22	0.06, ns
Folate (5.4–18 ng/mL)		-	13.38 ± 10.05	53.28 ± 82.4	53.28 ± 82.4	-	
TLC (4–11 × 10^9^/L)		6.1 ± 1.7	5.75 ± 3.17	4.05 ± 2.45	4.6 ± 2.96	3.53 ± 1.74	0.11, ns
Platelet (150–400 × 10^9^/L)		183 ±148	176 ± 161	56.64 ± 41.68	62.56 ± 51.46	50.93 ± 29.26	0.311, ns
Bilirubin (0.3–1.2 mg/dL)		1 ± 0.6	1.78 ± 1.55	4.21 ± 5.9	5.83 ± 7.64	2.53 ± 2.45	0.037 *
AST (5–40 IU/L)		35.3 ± 19.9	40.33 ± 13.82	63.33 ± 46.15	77.14 ± 56.86	49 ± 25.4	0.022 *
ALT (10–40 IU/L)		39.2 ± 10.8	26.17 ± 7.55	33.09 ± 19.24	33.17 ± 19.32	33 ± 19.52	0.974, ns
Albumin (3.5–5.2 g/dL)		4.5 ± 0.3	3.4 ± 0.87	3.1 ± 0.65	3.07 ± 0.62	3.13 ± 0.68	0.721, ns
INR		1.2 ± 0.2	1.36 ± 0.3	3.77 ± 15.55	1.92 ± 0.64	1.45 ± 0.36	0.001 *
Creatinine (0.2–1 mg/dL)		0.8 ± 0.1	0.71 ± 0.19	1.17 ± 0.99	1.21 ± 1.81	1.13 ± 0.76	0.763, ns

Values are expressed as mean ± SD. * *p* < 0.05, *** *p* < 0.001 compared with control group.

**Table 2:** Comparison of erythroid progenitor counts in bone marrow (BM) and peripheral blood (PB).

	Control	NCPF	Cirrhosis	Etiology-Based Division of Patients with Cirrhosis		
				Alcoholics	NASH	*p*-Value
Bone Marrow						
CD34+ (%)	9.16 ± 4.11	9.8 ± 4.85	6.56 ± 3.61	5.23 ± 2.86	6.34 ± 3.55	0.00426 *
EEP (%)	2.6 ± 1.8	9.07 ± 6.99	8.33 ± 10.7	7.34 ± 8.63	9.3 ± 12.49	0.48217
IEP (%)	77.6 ± 4.32	52.68 ± 14.15	58.5 ± 17.16	56.2 ± 14.47	61.47 ± 19.36	0.2386
LEP (%)	4.48 ± 0.4	12.77 ± 5.45	11.5 ± 9.5	13.32 ± 11.07	9.61 ± 7.43	0.1327
Peripheral Blood						
IEP (%)	0.16 ± 0.03	-	0.88 ± 0.46	-	-	0.026 *
LEP (%)	30.5 ± 12.2	-	59 ± 29.5	-	-	0.1078
sTfr1 (ng/mL)	2772 ± 172	2677 ± 428	2174 ± 470	2069 ± 431	2335 ± 411	0.022 *

* *p*-value significance < 0.05, Values are presented as mean ± SD.

## Data Availability

The original contributions presented in this study are included in the article/Supplementary Material. Further inquiries can be directed to the corresponding authors.

## Supplementary Materials

The following supporting information can be downloaded at: https://www.mdpi.com/article/10.3390/cimb48050511/s1.
