# Supplementary material for: Imperfect Maturation of Erythroid Progenitors in Patients with Cirrhosis-Associated Anemia
Source: Curr Issues Mol Biol. 2026 May 14;48(5):511. doi: 10.3390/cimb48050511 (PMC13204656; doi:10.3390/cimb48050511)
Supplement: Supplementary file 1 [file cimb-48-00511-s001.zip › Supplementary figures.pdf]

Supplementary Figures:

Supplementary figure S1:

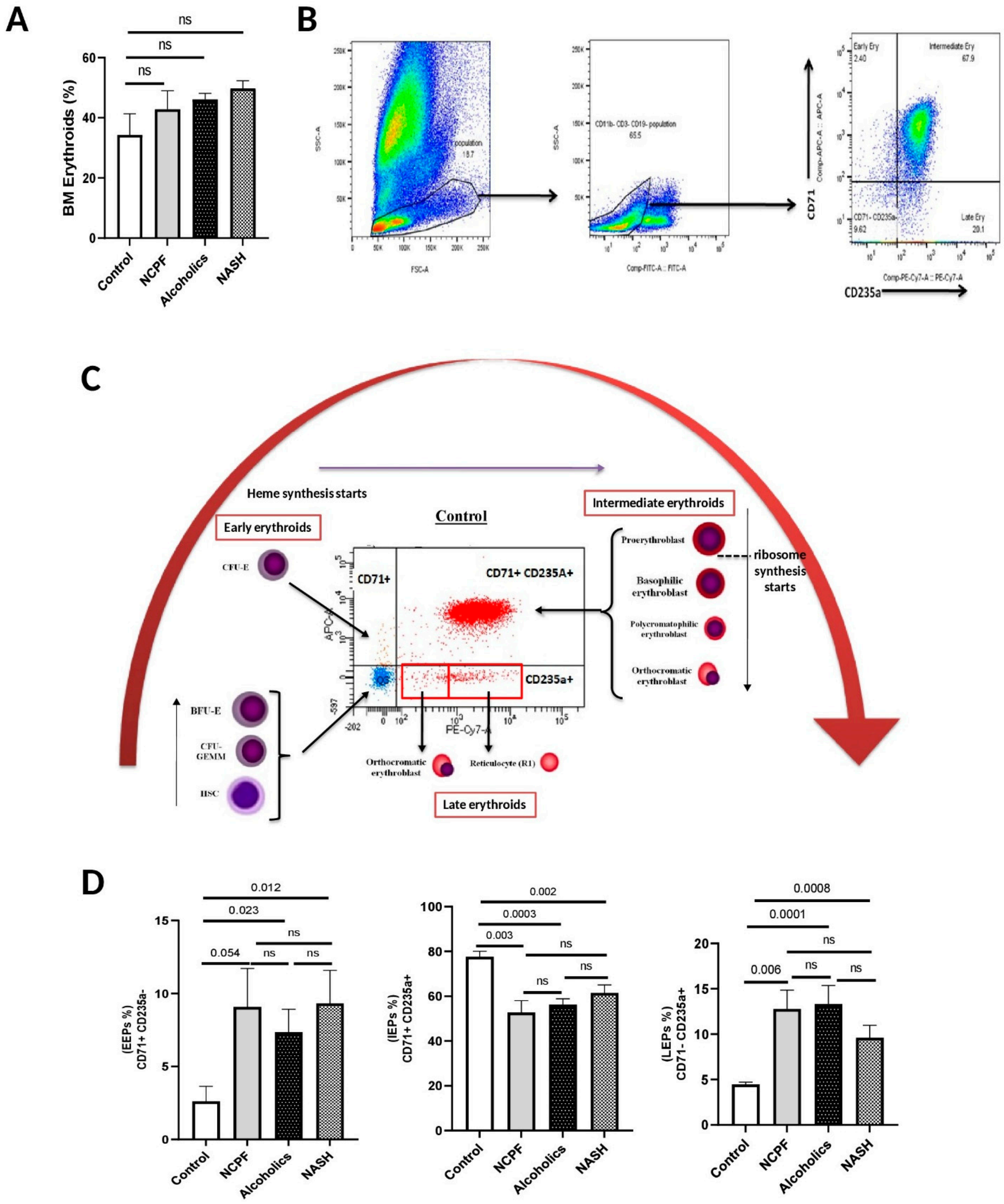

E

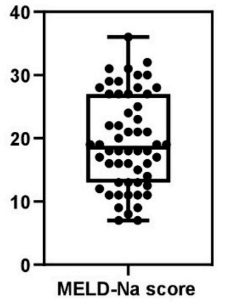

	MELD-Na score	No. of patients
Child A	0 -12	16
Child B	12 – 27	33
Child C	>27	11

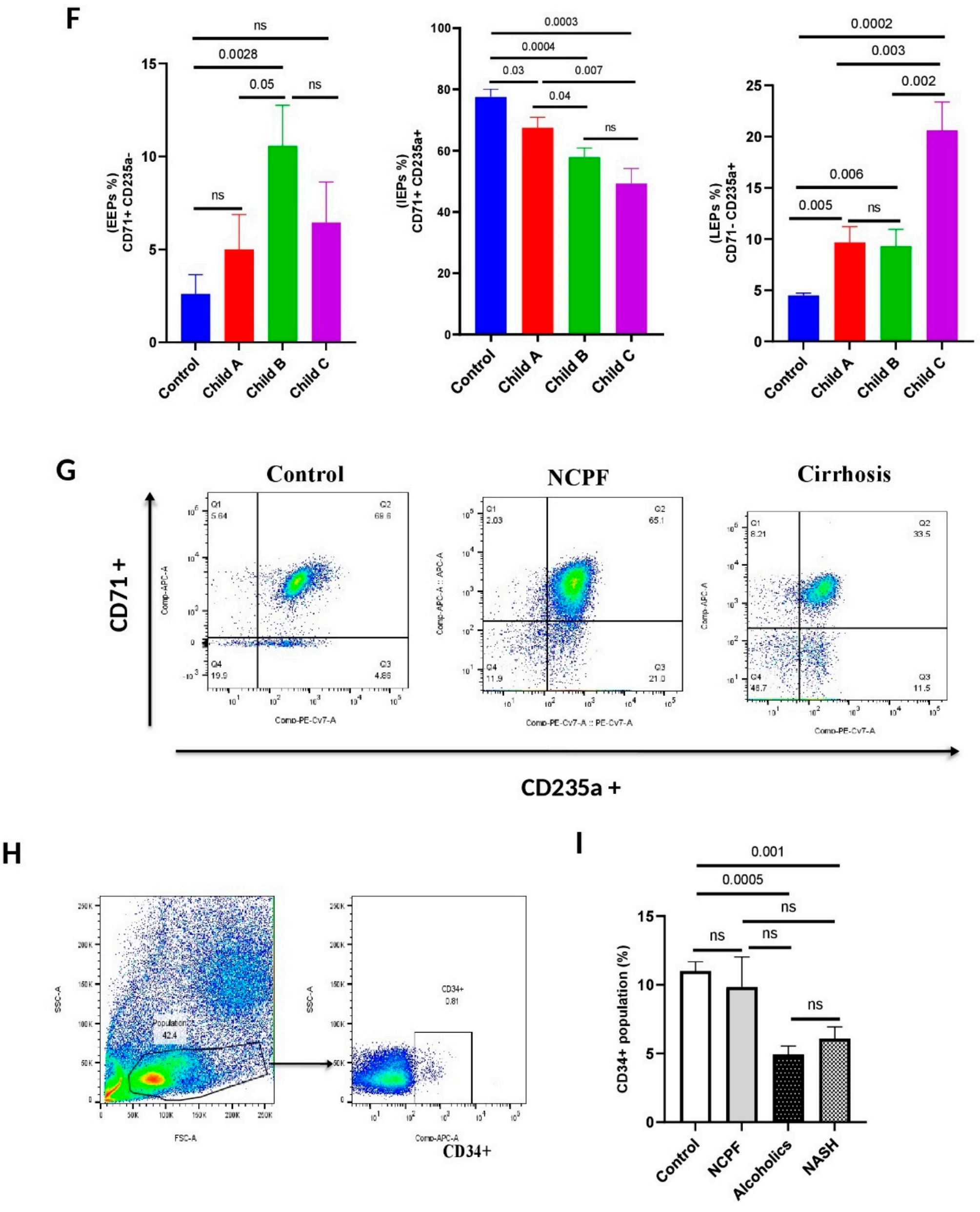

Figure S1: Total no. of erythroid cells (%) in BM of cirrhosis (n=60), alcoholics (n=30), NASH (n=30), NCPF (n=7) and control (n=3). (B) FACS data was analysed for total erythroblast population (%) using flowjo software in a dot plot. Stem cell population was selected from whole BM population among which CD11b-, CD3-, CD19- negative population was selected, finally erythroid population was characterized using CD71+ & CD235a+ antibodies. FACS graph representing the distinct population containing different stages of erythroid cells based on the expression of CD71(TfR1) and CD235a (Glycophorin A) markers. On an etiology basis, erythroid cell population EEPs, IEPs & LEPs were analyzed in alcoholics (n=30), NASH (n=30), NCPF (n=7) and control (n=3). (E) Median box plot graph plotted based on MELD-Na score of patients with cirrhosis using Graph pad Prism software (left). No. of patients lying in each MELD-Na score category represented as Child A, Child B, and Child C (right). (F) Erythroid population (%) EEPs, IEPs & LEPs were analyzed in cirrhosis (n=60), and control (n=3) in 3 different categories of MELD-Na score as Child A, B & C. (G) Representation of FACS analyzed erythroid population in the dot plot. (H) Total CD34+ HSCs population (%) was analyzed in whole BM cells. (I) Statistical graph of the total CD34+ HSC population (%) on an etiology basis was analyzed in alcoholics (n=18), NASH (n=17), NCPF (n=5), and control (n=3). *Exact p-values are shown for significant comparisons, while 'ns' indicates non-significant differences.*

Supplementary figure S2:

A

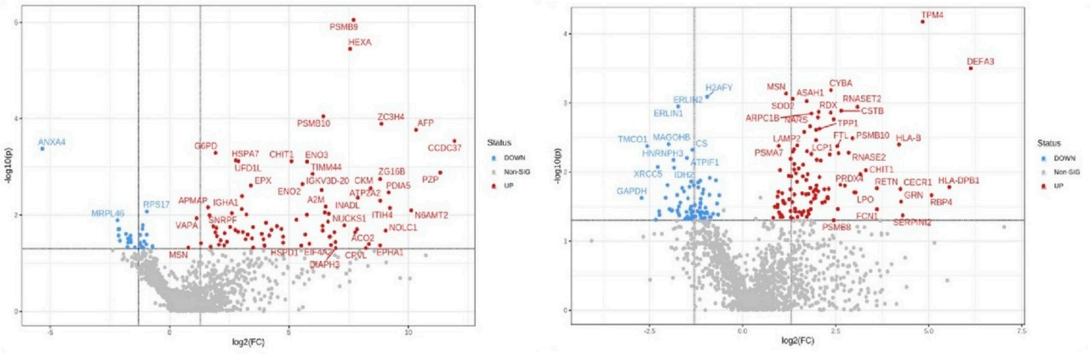

CLD Vs Control

CLD Vs NCPF

B

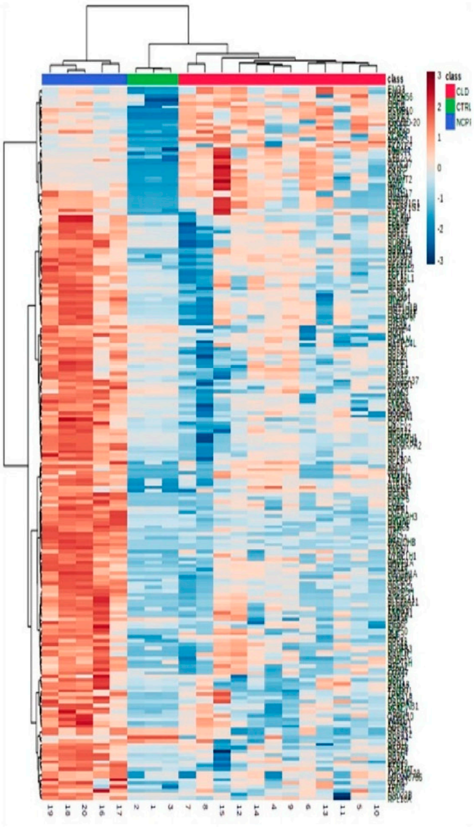

C

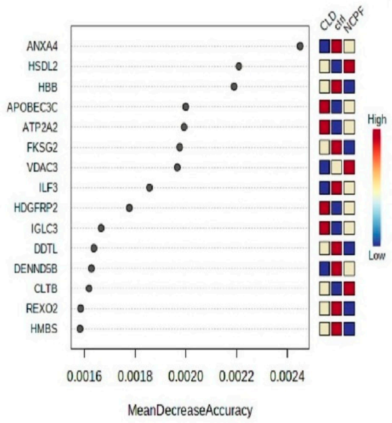

Supplimentary figure S2:

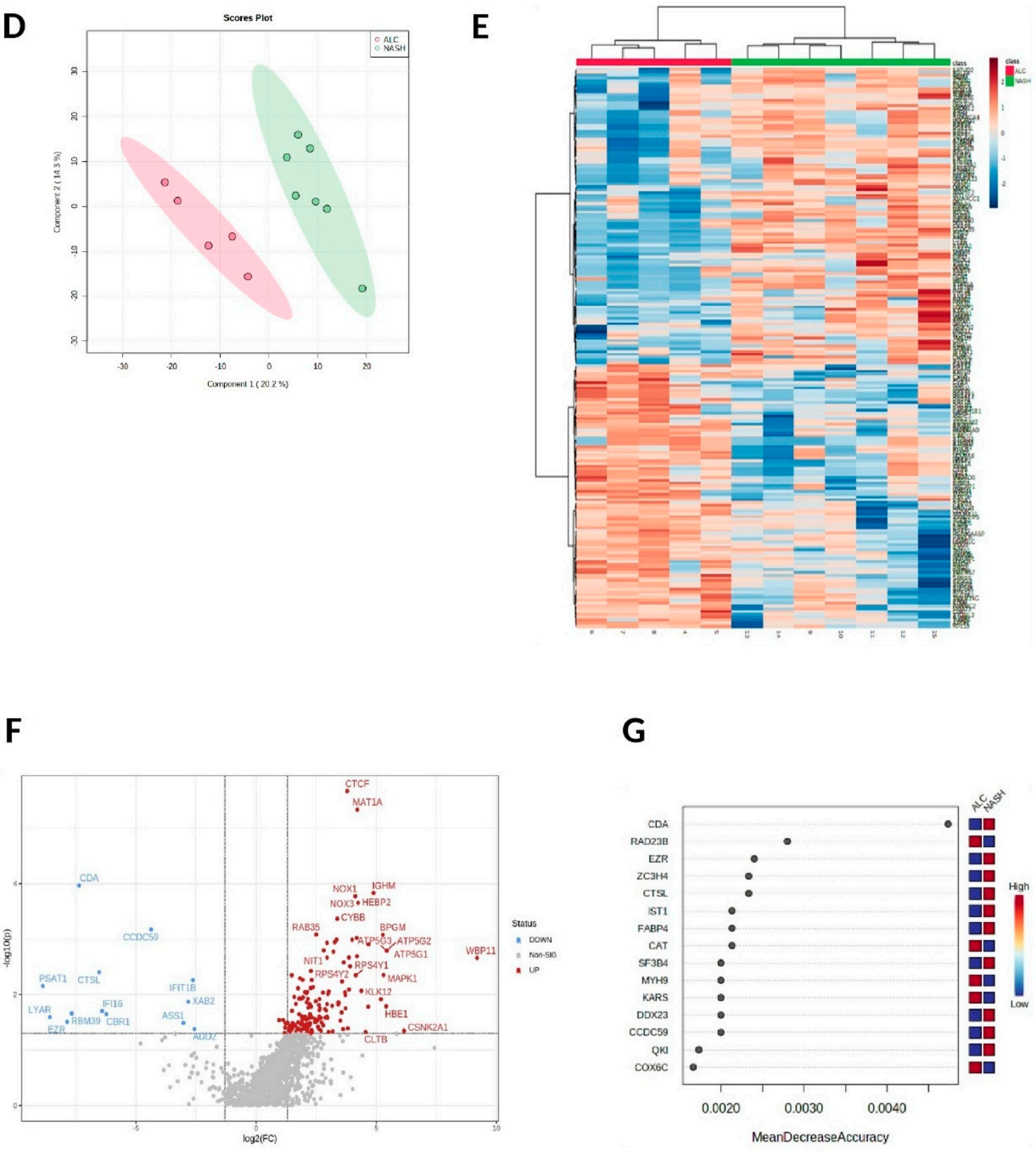

H

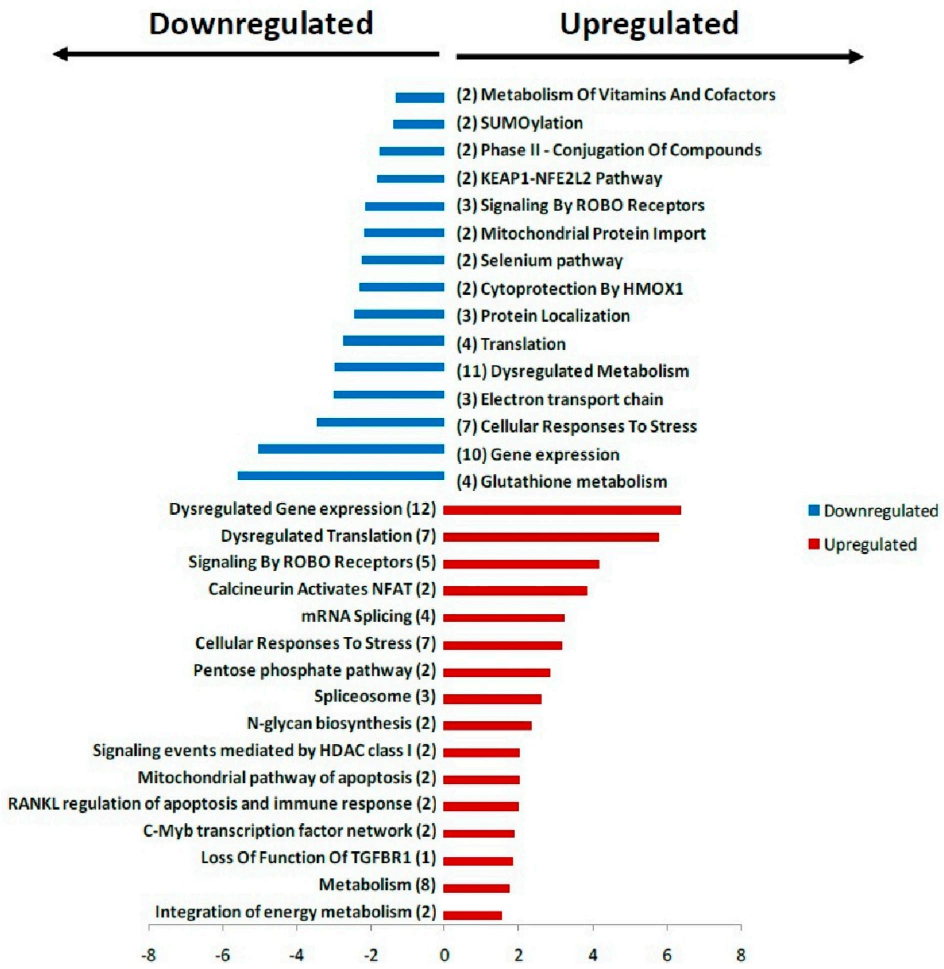

Figure S2: (A) Volcano plot of differentially expressed proteins (DEPs) from isolated CD71+ erythroid cells: CLD (n=12) versus control (n=3) (left) and CLD (n=12) versus NCPF (n=5). (B) Clustered heatmap of CD71+ erythroid cells representing a comparison of upregulated (Red) and downregulated (Blue) among CLD, NCPF and control groups. (C) Mean decrease accuracy plot. Mean decrease accuracy is the measure of the performance of the model without each protein. A higher value indicates the importance of CD71+ erythroid population specific protein in predicting group (CLD vs. NCPF vs. Control). (D) PLS-DA plot shows 2 distinct populations including alcoholic (n=5) [Pink] and NASH (n=7) [Green]. (E) Clustered heatmap of CD71+ erythroid population representing a comparison of upregulated (Red) and downregulated (Blue) between both groups. (F) Volcano plot of DEPs from isolated CD71+ erythroid population, alcoholic (n=5) versus NASH (n=7). **(G)** Mean Decrease Accuracy plot indicating CD71+ erythroid population specific protein in predicting group (alcoholics vs. NASH). **(H)** Upregulated (Red) and downregulated (Blue) pathways drawn from differentially expressed proteins (DEPs) in alcoholic (n=5). Reverse of these pathways means Downregulated (Red) and Upregulated (Blue) for NASH, (n=7).

Supplementary figure S3:

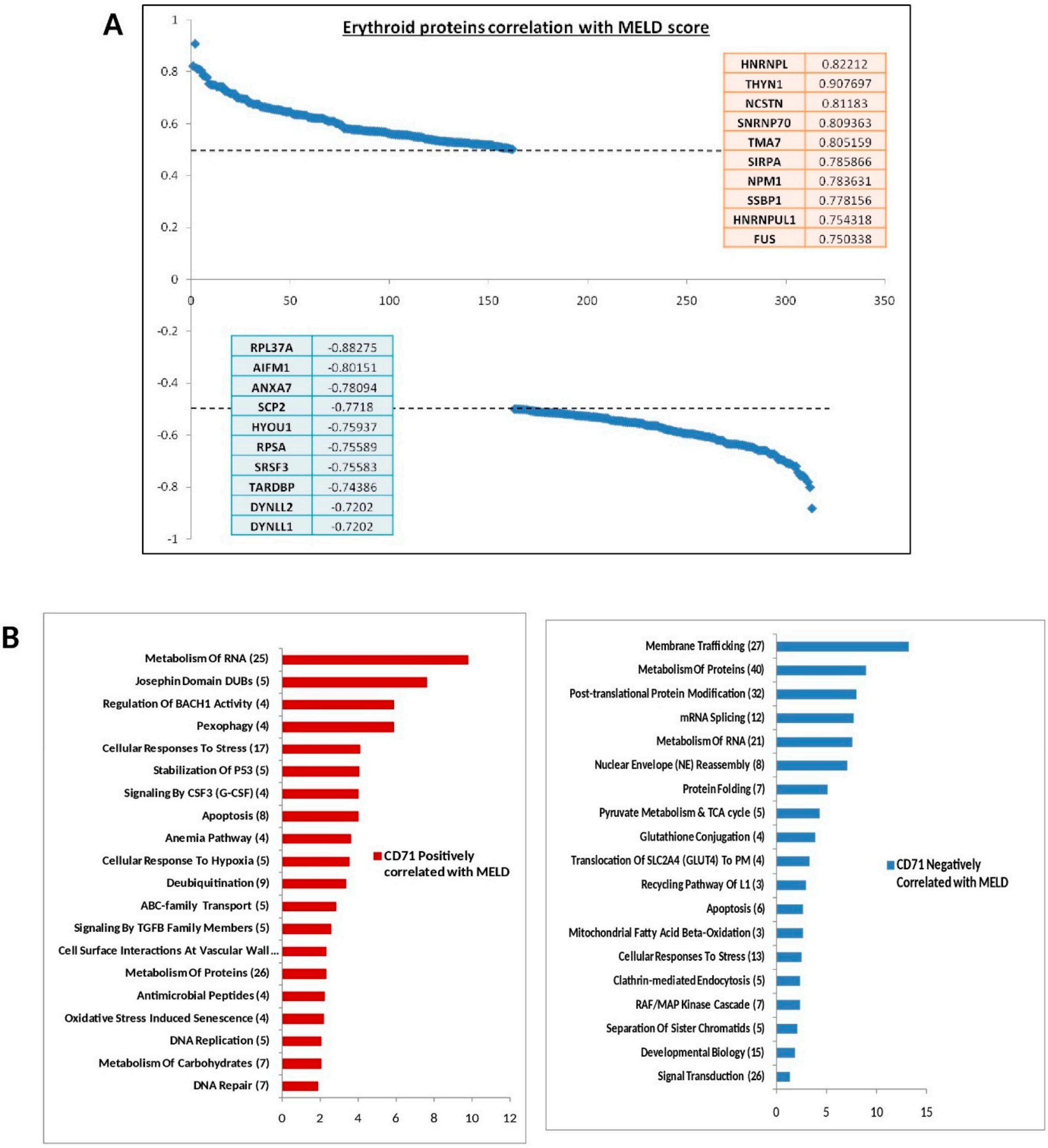

Supplementary figure S3:

Main Pathways	Sub-pathways	CLD	NCPF	CTRL
Heme biosythesis	Heme biosynthesis	-0.12068	0.119362	0.283802
	Hemoglobin complex	-0.11993	0.001811	0.476705
	Abnormal hemoglobin	-0.1262	0.257081	0.076315
	Persistence of F globin	-0.1356	0.314848	0.017637
Glutathione activity	Glutathione conjugation	0.004754	-0.05716	0.076255
	Glutathione metabolism	-0.0119	0.016202	0.020582
	Glutathione transferase activity	-0.04584	0.135572	-0.04258
Translation	Cytoplasmic translation	-0.30195	0.285439	-0.01578
	Positive regulation of translation in response to stress	-0.05647	0.093109	0.070679
	Translation factors	-0.08109	0.115379	0.132053
	Post translational modification	-0.08125	0.016713	0.297144
	Pre-initiation complex	0.001635	0.077509	-0.13572
	Check points signal	-0.05215	0.191199	-0.11006
	Mitochondrial translation	-0.13025	0.098868	0.356234
Cell cycle	Negative regulation of cell cycle G2 M phase transition	-0.02058	0.243865	-0.32413
	Positive regulation of cell cycle G2 M phase transition	0.018516	-0.08243	0.063325
	Regulation of mitotic cell cycle phase transition	0.012188	-0.04033	0.018457
	TP53 regulates transcription of cell cycle genes	-0.00817	-0.12488	0.240835
	M phase of mitotic cell cycle	0.012404	0.266247	-0.49336
Mitochondrial activity	Mitochondrial DNA replication	0.002613	-0.16542	0.265244
	Mitochondrial electron transport cytochrome C to O2	-0.12249	0.179125	0.191409
	Mitochondrial electron transport NADH to Ubiquitin	-0.0589	0.186407	-0.07509
	Mitochondrial fatty acid beta oxidation	-0.04828	0.236651	-0.20129
Apoptosis	Intrinsic pathway of apoptosis	0.027818	-0.25717	0.317354
	Apoptosis by reversed IL-6	0.046482	-0.10714	-0.00736
	Apoptosis via TRAIL up	-0.00205	-0.01681	0.036222
	Hallmark of apoptosis	0.02512	-0.14352	0.13872
	Regulation of apoptosis	0.015465	-0.05199	0.024785
	Suppression of apoptosis	-0.01929	-0.23353	0.466366
Erythropoiesis	Erythroid hyperplasia	-0.23622	0.271481	0.492406
	Abnormal no. Of erythroid precursor	-0.13856	0.301085	0.052417
	Erythroid differentiation	-0.02618	0.095178	-0.05392

Figure S3: (A) Correlation graph between CD71+ erythroid cells (n=12) and MELD-Na score with significance cut-off value of above +0.5 for upregulated proteins and below -0.5 for downregulated proteins. (B) Pathways positively correlated with MELD-Na score in CD71+ erythroid cells (in red bars). Pathways negatively correlated with MELD-Na score in CD71+ erythroid cells (in blue bars). (C) Log2 fold-change (log2FC) values

of key erythroid pathways (heme biosynthesis, glutathione activity, translation, cell cycle, mitochondrial activity, apoptosis, and erythropoiesis) across Control, NCPF, and CLD groups. Positive values indicate upregulation and negative values indicate downregulation.

Supplementary figure 4(A-C)

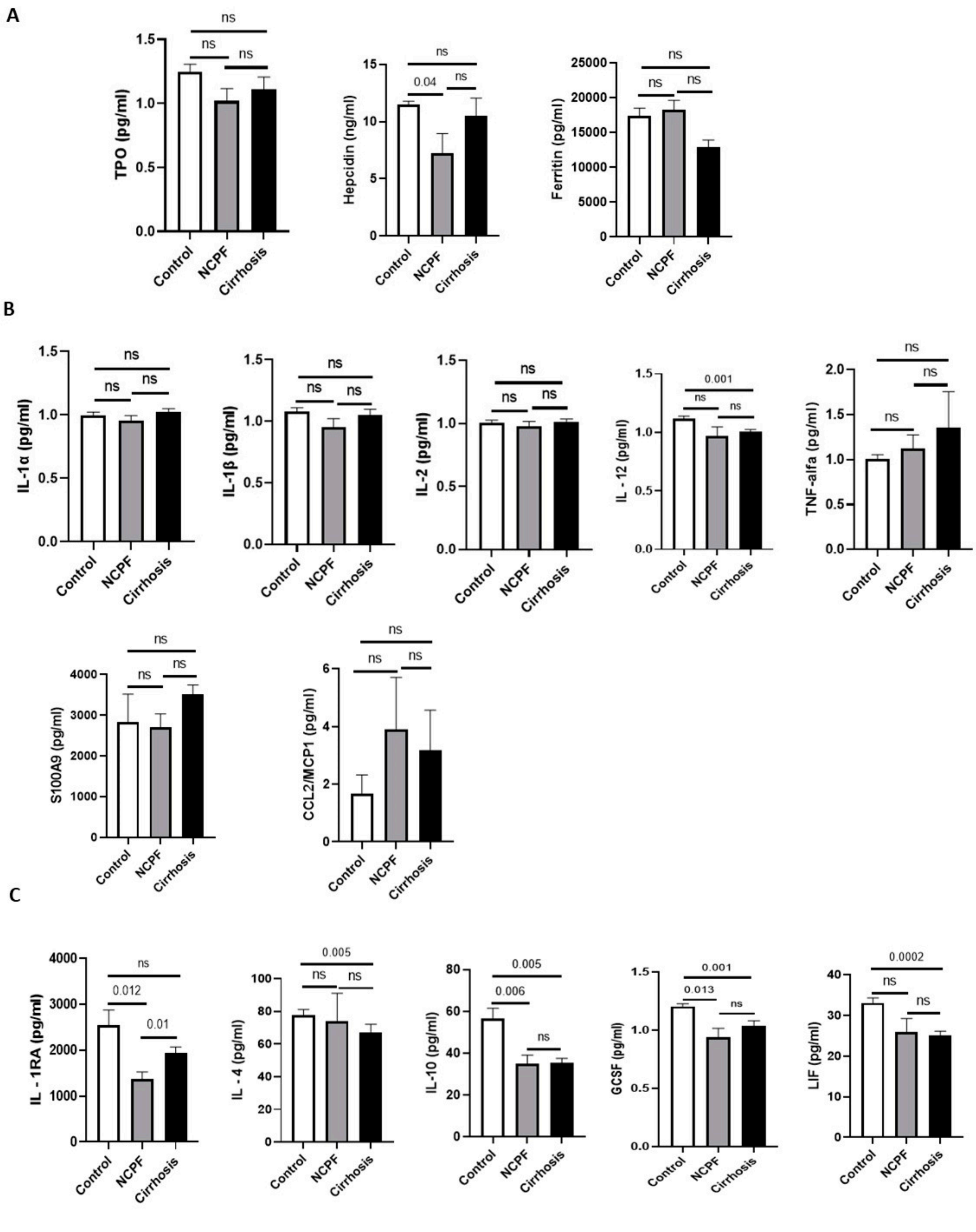

Figure S4: Expression of (A) erythropoiesis regulating agents: TPO, Hepcidin, and Ferritin, (B) Pro-inflammatory cytokines: IL-1α, IL-1β, IL-2, IL-12, TNF-α, S100-A9 and CCL2/JE/MCP-1, (C) IL-1Ra, IL-10, GCSF and LIF in cirrhosis as compared to NCPF and control.). *Exact p-values are shown for significant comparisons, while 'ns' indicates non-significant differences.*
