# Supplementary material for: Imperfect Maturation of Erythroid Progenitors in Patients with Cirrhosis-Associated Anemia
Source: Curr Issues Mol Biol. 2026 May 14;48(5):511. doi: 10.3390/cimb48050511 (PMC13204656; doi:10.3390/cimb48050511)
Supplement: Supplementary file 1 [file cimb-48-00511-s001.zip › Supplementary tables.pdf]

Table S1: Spearman correlation of EEPs, IEPs, and LEPs with hemoglobin (hb) and severity of disease in patients with cirrhosis. Statistical significance is indicated as follows: $p < 0.05$ (*), $p < 0.01$ (**), $p < 0.001$ (***), $p < 0.0001$ (****); ns = not significant

	Hb vs. HCT	Hb vs. MCV	Hb vs. RDW	Hb vs. RBC	Hb vs. Na-MELD	Hb vs. BMerythroid no.	Hb vs. Total CD71+	Hb vs. CD71- CD235a-	[EEP] Hb vs. CD71+ CD235-	[IEP] Hb vs. CD71+ CD235+	[LEP] Hb vs. CD71- CD235+
Spearman r											
r	0.9779	0.02722	-0.1681	0.8543	-0.2812		0.888	0.261	0.056	-0.691	-0.807
P (two-tailed)	<0.0001	0.7017	0.1792	<0.0001	0.0421	0.509	0.003	0.294	0.962	0.024	0.009
P value summary	****	ns	ns	****	*	ns	**	ns	ns	*	**
Significant? (alpha = 0.05)	Yes	No	No	Yes	Yes	No	Yes	No	No	Yes	Yes
Number of XY Pairs	53	53	53	53	53	53	53	53	53	53	53

	Na-MELD vs. Hb	Na-MELD vs. HCT	Na-MELD vs. MCV	Na-MELD vs. RDW	Na-MELD vs. RBC	Na-MELD vs. BMerythroid no.	Na-MELD vs. Total CD71+	Na-MELD vs. CD71- CD235a-	[EEP] Na-MELD vs. CD71+ CD235-	[IEP] Na-MELD vs. CD71+ CD235+	[LEP] Na-MELD vs. CD71- CD235+
Spearman r											
r	-0.2803	-0.3446	0.1312	0.1617	-0.3495	-0.1823	-0.5261	0.2984	0.2343	-0.4422	0.3865
95% confidence interval	-0.5178 to -0.002640	-0.5681 to -0.07384	-0.1523 to 0.3946	-0.1217 to 0.4206	-0.5718 to -0.07930	-0.4380 to 0.1006	-0.7015 to -0.2908	0.02236 to 0.5321	-0.04660 to 0.4809	-0.6413 to -0.1873	0.1217 to 0.6000
P (two-tailed)	0.0421	0.0115	0.3492	0.2475	0.0103	0.1913	<0.0001	0.0300	0.0913	0.0009	0.0043
P value summary	*	*	ns	ns	*	ns	****	*	ns	***	**
Significant? (alpha = 0.05)	Yes	Yes	No	No	Yes	No	Yes	Yes	No	Yes	Yes
Number of XY Pairs	53	53	53	53	53	53	53	53	53	53	53

Table S2: Representing the number of each colony in two different groups: control (n=3) and cirrhosis (n=4).

Colony forming unit (CFU) assay					
	Colonies count				
**Subjects**	**Total**	**CFU – E**	**BFU - E**	**CFU - GM**	**CFU - GEMM**
**Control 1**	79	4	59	9	7
**Control 2**	82	5	57	14	6
**Control 3**	89	3	69	17	3
**Average ± S.E.**	**84.3 ± 3.9**	**4 ± 0.6**	**61.7 ± 3.7**	**13.3 ± 2.3**	**5.3 ± 1.2**
**Patient 1**	59	2	37	17	3
**Patient 2**	41	3	30	5	3
**Patient 3**	24	2	13	8	1
**Patient 4**	25	2	18	4	1
**Average ± S.E.**	**37.25 ± 8.2**	**2.25 ± 0.3**	**24.5 ± 5.5**	**8.5 ± 3**	**2 ± 06**

Table S3: List of DEPs of CD71+ erythroid cells – CLD versus control. FC represents fold change.

Up-regulated proteins							
**Protein**	**FC**	**log2(FC)**	**raw.pval**	**Protein**	**FC**	**log2(FC)**	**raw.pval**
**MSN**	1.719	0.78153	0.047674	**FN1**	11.279	3.4955	0.017839
**VAPA**	2.1833	1.1265	0.011656	**ALDH3A2**	11.315	3.5002	0.047538
**HSPA5**	2.4913	1.3169	0.0385	**SPTBN1**	14.447	3.8527	0.024439
**APMAP**	3.0574	1.6123	0.006906	**UBE2L3**	15.274	3.933	0.031736
**SNRPF**	3.1821	1.67	0.010084	**RPL7A**	15.678	3.9707	0.04238
**LCP1**	3.3022	1.7234	0.045428	**NOX1**	15.707	3.9734	0.043549
**LAP3**	3.5487	1.8273	0.017048	**NOX3**	15.707	3.9734	0.043549
**ATP1A2**	3.786	1.9207	0.022821	**ACTA1**	16.424	4.0378	0.014377
**G6PD**	3.8068	1.9286	0.00051	**ACTA2**	16.424	4.0378	0.014377
**IGLC2**	3.9048	1.9652	0.01854	**ACTC1**	16.424	4.0378	0.014377
**IGLC3**	3.9048	1.9652	0.01854	**ACTG2**	16.424	4.0378	0.014377
**CREG1**	3.952	1.9826	0.027266	**RPS20**	18.037	4.1729	0.026547
**GALK1**	4.1924	2.0678	0.041194	**ENO1**	19.265	4.2679	0.020165
**PLS3**	4.3946	2.1357	0.033391	**BPIFB1**	22.523	4.4933	0.016942
**ACTB**	4.8659	2.2827	0.042518	**YARS**	25.713	4.6844	0.019457
**ACTG1**	4.8659	2.2827	0.042518	**TMBIM1**	26.945	4.7519	0.028072
**ATP1A3**	4.9423	2.3052	0.023679	**SERPINC1**	26.985	4.7541	0.037636
**IGKV4-1**	5.2906	2.4034	0.035787	**CHIT1**	34.259	5.0984	0.000759
**IGLC1**	5.7986	2.5357	0.017673	**ELAVL1**	39.799	5.3147	0.012481
**ATP2A1**	6.0842	2.6051	0.009174	**HSPD1**	45.673	5.5133	0.043167
**PHB2**	6.7133	2.747	0.020262	**ENO2**	47.108	5.5579	0.00228
**HSPA7**	6.8157	2.7689	0.000733	**TKTL1**	51.214	5.6785	0.025428
**IMMT**	7.3088	2.8696	0.022315	**DNAH17**	53.704	5.747	0.009828
**UFD1L**	7.3767	2.883	0.000754	**DNAH9**	53.704	5.747	0.009828
**TMEM56**	8.1384	3.0247	0.007544	**ENO3**	54.143	5.7587	0.000776
**IGHA1**	8.1974	3.0352	0.004052	**EIF2S2**	57.79	5.8527	0.039424
**SERPINB6**	8.3677	3.0648	0.022511	**TIMM44**	63.483	5.9883	0.001401
**RPN2**	9.0853	3.1835	0.031631	**IGKV3D-20**	82.4	6.3646	0.003034
**TMPO**	9.2945	3.2164	0.009738	**RBP4**	84.72	6.4046	0.016762
**GNS**	9.6814	3.2752	0.025375	**ATP6V1G1**	84.967	6.4088	0.020919
**EPX**	10.559	3.4004	0.002432	**ATP6V1G2**	84.967	6.4088	0.020919

**PSMB10**	86.6	6.4363	8.94E-05
**AKR1E2**	90.01	6.492	0.008882
**A2M**	92.722	6.5348	0.006651
**ATAD3B**	98.962	6.6288	0.013665
**NUCKS1**	101.54	6.6659	0.009396
**MYO18A**	104.03	6.7008	0.028224
**GC**	105.33	6.7187	0.028041
**EIF4A2**	107.99	6.7547	0.041901
**SART1**	119.43	6.9	0.022715
**RPS7**	122.85	6.9408	0.036353
**DIAPH3**	123.87	6.9527	0.048864
**RAB25**	159.49	7.3173	0.016373
**HEXA**	189.13	7.5633	3.57E-06
**PSMB9**	208.57	7.7044	8.85E-07
**PPARD**	218.18	7.7694	0.02288
**IGHA2**	230.29	7.8473	0.019756
**INADL**	236.98	7.8886	0.004391
**CPVL**	298.52	8.2217	0.048772
**ACO2**	326.39	8.3504	0.039831
**CKM**	342.53	8.4201	0.002791
**EPHA1**	451.7	8.8192	0.04261
**ZG16B**	452	8.8202	0.001796
**ATP2A2**	453.07	8.8236	0.00502
**ZC3H4**	468.26	8.8712	0.000127
**NOLC1**	527.64	9.0434	0.020968
**PDIA5**	576.62	9.1715	0.003388
**ITIH4**	597.44	9.2226	0.007086
**N6AMT2**	1109.2	10.115	0.008003
**AFP**	1274	10.315	0.00017
**PZP**	2576.1	11.331	0.001309
**CCDC37**	3897.2	11.928	0.00029

Down-regulated proteins			
**Protein**	**FC**	**log2(FC)**	**raw.pval**
**ANXA4**	0.024836	-5.3314	0.00042
**MRPL46**	0.22108	-2.1773	0.012857
**FN3KRP**	0.22613	-2.1448	0.019615
**MAPK1**	0.22756	-2.1357	0.026583
**FGA**	0.23294	-2.102	0.019549
**HAGH**	0.23709	-2.0765	0.033822
**HBB**	0.2817	-1.8278	0.024441
**USP5**	0.29597	-1.7565	0.045941
**RPS4Y1**	0.29986	-1.7377	0.030791
**RPS4Y2**	0.29986	-1.7377	0.030791
**CCS**	0.30037	-1.7352	0.037278
**ALAD**	0.32285	-1.6311	0.02683
**RPS4X**	0.32455	-1.6235	0.03266
**HPRT1**	0.32651	-1.6148	0.036728
**RPA1**	0.36676	-1.4471	0.046852
**HNRNPL**	0.42347	-1.2397	0.03774
**CA1**	0.42648	-1.2294	0.026028
**RBM8A**	0.43983	-1.185	0.044453
**PGAM2**	0.45558	-1.1342	0.019343
**HSP90AA2**	0.4599	-1.1206	0.016212
**RALY**	0.46565	-1.1027	0.047807
**PRDX2**	0.47573	-1.0718	0.03497
**GDI2**	0.51056	-0.96986	0.040474
**TRA2A**	0.51532	-0.95647	0.025596
**RPS17**	0.51673	-0.95253	0.008404
**HIST2H2AB**	0.60842	-0.71686	0.044684

Table S4: List of DEPs of CD71+ erythroid cells– CLD versus NCPF. FC represents fold change.

Up-regulated proteins							
Protein	FC	log2(FC)	raw.pval	Protein	FC	log2(FC)	raw.pval
**IGHG1**	1.7642	0.81904	0.045952	**APMAP**	2.4421	1.2881	0.006444
**ACTN4**	1.8104	0.8563	0.031846	**ISYNA1**	2.4548	1.2956	0.045432
**SEPTIN12**	1.908	0.93209	0.032359	**MCTS1**	2.4711	1.3051	0.011528
**TXN**	1.9113	0.93457	0.022548	**DNAJC7**	2.5253	1.3365	0.027185
**TPI1**	1.915	0.93731	0.029708	**SOD2**	2.5437	1.3469	0.000872
**RAP1BL**	1.9453	0.95996	0.019399	**CTSD**	2.5467	1.3486	0.005063
**PSMA7**	1.957	0.96863	0.004176	**GIGYF2**	2.5577	1.3548	0.0478
**HNRNPA1L2**	1.966	0.97527	0.038284	**C11orf54**	2.5968	1.3767	0.004702
**RAP1B**	2.0062	1.0044	0.009369	**MDH1**	2.6048	1.3812	0.009168
**PPIB**	2.007	1.0051	0.041143	**CTNNA1**	2.6073	1.3825	0.00763
**TALDO1**	2.0183	1.0131	0.044692	**B2M**	2.6145	1.3865	0.021185
**NME1**	2.0572	1.0407	0.042007	**ATP2A1**	2.6457	1.4036	0.0338
**PGAM1**	2.0876	1.0618	0.020216	**TKT**	2.67	1.4168	0.037342
**TPM3**	2.1021	1.0718	0.044585	**ATL3**	2.7009	1.4335	0.010415
**RBM4**	2.1157	1.0812	0.025987	**HSPA1B**	2.7489	1.4588	0.013794
**RBM4B**	2.1157	1.0812	0.025987	**USP7**	2.76	1.4646	0.004112
**M6PR**	2.1353	1.0945	0.027339	**CSTF3**	2.7739	1.4719	0.044585
**NME2P1**	2.1845	1.1273	0.028481	**LAP3**	2.8401	1.506	0.008877
**MSN**	2.2426	1.1652	0.000733	**MMP8**	2.8718	1.522	0.037827
**PGAM4**	2.3559	1.2363	0.011344	**FAH**	3.0148	1.5921	0.041416
**NME2**	2.3775	1.2495	0.024757	**CTSG**	3.0338	1.6011	0.020325
**ITGB2**	2.4369	1.285	0.017808	**CAP2**	3.0654	1.6161	0.011559
**LYZ**	2.4385	1.286	0.036599	**FKBP5**	3.0665	1.6166	0.038207

Protein	FC	log2(FC)	raw.pval	Protein	FC	log2(FC)	raw.pval
**LMAN2**	3.0786	1.6223	0.007835	**SIX5**	4.5686	2.1917	0.017749
**MOGS**	3.1102	1.637	0.036952	**COPA**	4.6324	2.2118	0.026936
**LAMP2**	3.1389	1.6502	0.002637	**PRTN3**	4.8211	2.2694	0.023869
**PLS3**	3.2548	1.7026	0.021157	**CISD1**	4.8555	2.2796	0.022953
**AIMP2**	3.2632	1.7063	0.013001	**BPI**	4.9052	2.2943	0.02399
**ASAH1**	3.2891	1.7177	0.000941	**ACTN1**	4.9665	2.3122	0.017542
**ELANE**	3.2941	1.7199	0.044479	**C19orf10**	5.0597	2.339	0.00555
**WDR1**	3.3095	1.7266	0.00499	**VCL**	5.1482	2.3641	0.001384
**CPNE3**	3.359	1.748	0.010735	**CYBA**	5.177	2.3721	0.000651
**ROCK1**	3.3962	1.7639	0.03655	**SERPINB1**	5.4478	2.4457	0.001722
**ARHGDIA**	3.407	1.7685	0.020838	**PSMB8**	5.4541	2.4473	0.049642
**AZU1**	3.4195	1.7738	0.040522	**FTL**	5.8052	2.5374	0.004222
**NARS**	3.5038	1.8089	0.002183	**S100A14**	5.8864	2.5574	0.034358
**MPO**	3.5255	1.8178	0.017048	**TMOD3**	5.9693	2.5776	0.005352
**CNBP**	3.5484	1.8272	0.034616	**NPC2**	6.1588	2.6227	0.015304
**LOC100128009**	3.5734	1.8373	0.027285	**CSTB**	6.2649	2.6473	0.001302
**CLC**	3.599	1.8476	0.005472	**CEACAM6**	6.7152	2.7474	0.015754
**ARPC1B**	3.6066	1.8506	0.001427	**RNASE2**	7.1859	2.8452	0.005254
**GUSB**	3.7273	1.8981	0.01925	**PSMB10**	7.7401	2.9524	0.003253
**GLUL**	3.7548	1.9087	0.024314	**GNS**	7.9745	2.9954	0.019705
**ARHGDIB**	3.8545	1.9465	0.018049	**LPO**	8.2112	3.0376	0.019764
**GSTP1**	3.8603	1.9487	0.023772	**RNASET2**	8.4862	3.0851	0.001142
**CREG1**	3.866	1.9508	0.021713	**PRDX4**	8.9402	3.1603	0.010738
**LCP1**	3.9186	1.9703	0.003463	**CHIT1**	9.8925	3.3063	0.009414
**AIMP1**	3.9204	1.971	0.018038	**RETN**	12.198	3.6085	0.017225
**IQGAP1**	3.9233	1.9721	0.002456	**FCN1**	12.214	3.6105	0.034649
**PSMA4**	3.9539	1.9833	0.015724	**HLA-B**	18.365	4.1989	0.004002
**CAP1**	3.9696	1.989	0.006658	**CECR1**	18.907	4.2408	0.017599
**ESYT1**	4.0593	2.0212	0.001622	**GRN**	19.12	4.257	0.026834
**CD63**	4.0755	2.027	0.019088	**SERPINI2**	19.71	4.3008	0.042572
**RDX**	4.1156	2.0411	0.001345	**TPM4**	28.578	4.8369	6.68E-05
**PSMB9**	4.1769	2.0624	0.011834	**RBP4**	33.721	5.0756	0.021639
**TPP1**	4.1866	2.0658	0.002368	**HLA-DPB1**	46.936	5.5526	0.016522
**PARK7**	4.2794	2.0974	0.006812	**DEFA3**	69.992	6.1291	0.000316

Down-regulated proteins							
**Protein**	**FC**	**log2(FC)**	**raw.pval**	**Protein**	**FC**	**log2(FC)**	**raw.pval**
**GAPDH**	0.15201	-2.7178	0.023707	**H3F3A**	0.34369	-1.5408	0.047606
**TMCO1**	0.16879	-2.5667	0.004216	**TPD52L2**	0.35063	-1.512	0.037859
**CCAR2**	0.19815	-2.3354	0.048785	**ATPIF1**	0.35352	-1.5001	0.006236
**XRCC5**	0.20547	-2.283	0.008444	**H1F0**	0.35635	-1.4886	0.043969
**RPL22**	0.21078	-2.2462	0.036979	**RPL13**	0.36042	-1.4723	0.047041
**ERP29**	0.21204	-2.2376	0.041792	**SRSF5**	0.36111	-1.4695	0.023988
**CLTCL1**	0.23807	-2.0705	0.023613	**RPS18**	0.37059	-1.4321	0.016371
**HIST1H1A**	0.24627	-2.0217	0.015474	**MPDU1**	0.37671	-1.4085	0.034382
**MAGOHB**	0.25154	-1.9911	0.003966	**SLC25A37**	0.37705	-1.4072	0.046678
**TUBB1**	0.25515	-1.9706	0.034446	**RPS19**	0.38413	-1.3803	0.031491
**HIST1H1E**	0.26592	-1.9109	0.036086	**ATP1A3**	0.38518	-1.3764	0.048141
**SEC61B**	0.26862	-1.8963	0.035831	**CTSL**	0.38538	-1.3756	0.039225
**HNRNPH3**	0.2753	-1.8609	0.006679	**HNRNPM**	0.38793	-1.3661	0.043268
**IDH2**	0.27849	-1.8443	0.008656	**PSMD1**	0.38963	-1.3598	0.036873
**EIF3G**	0.29503	-1.7611	0.021504	**CS**	0.39126	-1.3538	0.004781
**FKBP8**	0.29694	-1.7518	0.035872	**ST13**	0.39693	-1.333	0.04459
**HIST1H1T**	0.29967	-1.7386	0.038694	**ST13P4**	0.39693	-1.333	0.04459
**ERLIN1**	0.30045	-1.7348	0.001122	**ST13P5**	0.39693	-1.333	0.04459
**ERLIN2**	0.30045	-1.7348	0.001122	**PSMC5**	0.40082	-1.319	0.015534
**RPL7A**	0.3045	-1.7155	0.031605	**RPL5**	0.41043	-1.2848	0.030012
**RPS14**	0.30732	-1.7022	0.042611	**DDOST**	0.41508	-1.2685	0.043815
**RPS15**	0.31241	-1.6785	0.027678	**RPL3**	0.42975	-1.2184	0.036899
**DDX18**	0.32436	-1.6243	0.017024	**RPL4**	0.43558	-1.199	0.014198
**RNPS1**	0.33487	-1.5783	0.019106	**SLC25A21**	0.43744	-1.1929	0.016631
**FKBP3**	0.3431	-1.5433	0.047845	**UQCRC1**	0.44094	-1.1814	0.037936
**HIST1H3F**	0.34342	-1.5419	0.047619	**RPL27**	0.44886	-1.1557	0.032657
**HIST2H3A**	0.34342	-1.5419	0.047619	**RPL7**	0.45327	-1.1416	0.013356
**HIST3H3**	0.34365	-1.541	0.047672	**DDX50**	0.4542	-1.1386	0.041788

Protein	FC	log2(FC)	raw.pval
**PCBP1**	0.45613	-1.1325	0.027916
**NUP50**	0.45717	-1.1292	0.028726
**DEK**	0.45808	-1.1263	0.02435
**HNRNPH1**	0.45891	-1.1237	0.047444
**HNRNPH2**	0.45891	-1.1237	0.047444
**CHMP6**	0.46531	-1.1037	0.037715
**RPS5**	0.47378	-1.0777	0.020395
**SCCPDH**	0.47621	-1.0703	0.046501
**RPL30**	0.4813	-1.055	0.01797
**ELOVL5**	0.48912	-1.0317	0.039615
**TRIM28**	0.49548	-1.0131	0.045981
**RAB35**	0.49861	-1.004	0.032779
**RPL26**	0.51206	-0.96562	0.03794
**H2AFY**	0.51538	-0.95628	0.000814
**ZFPM1**	0.51671	-0.95259	0.039698
**RPS8**	0.53896	-0.89176	0.0214
**RPL26L1**	0.54068	-0.88716	0.044989
**RPL6**	0.54194	-0.88379	0.012148
**RPS9**	0.55201	-0.85724	0.045039
**COX2**	0.57607	-0.79567	0.040113
**VDAC3**	0.60756	-0.71891	0.013207
**HDGF**	0.61902	-0.69193	0.028673
**VAPA**	0.63312	-0.65946	0.04123
**HNRNPU**	0.64578	-0.63089	0.0217

Table S5: List of DEPs of CD71+ erythroid cells – Alcoholic versus NASH. FC represents fold change.

Down-regulated proteins							
**Protein**	**FC**	**log2(FC)**	**raw.pval**	**Protein**	**FC**	**log2(FC)**	**raw.pval**
**RBM39**	0.00124	-9.6557	0.005884	**SAFB2**	0.18282	-2.4515	0.019826
**EZR**	0.00168	-9.217	0.021583	**PRPH**	0.18668	-2.4214	0.032646
**PSAT1**	0.001796	-9.1214	7.24E-05	**PSMC5**	0.18902	-2.4034	0.002828
**CBR1**	0.002084	-8.9063	0.006766	**LDHA**	0.1966	-2.3467	0.014006
**LYAR**	0.003215	-8.281	0.000596	**EPDR1**	0.20163	-2.3102	0.037598
**CDA**	0.004009	-7.9627	2.65E-07	**FABP4**	0.20349	-2.297	0.021908
**IFI16**	0.007854	-6.9924	0.000587	**SLC25A1**	0.21108	-2.2441	0.046074
**CTSL**	0.008891	-6.8134	2.67E-06	**SPCS3**	0.22085	-2.1788	0.004555
**VPS4B**	0.012458	-6.3268	0.025653	**CKAP5**	0.23008	-2.1198	0.005416
**SNX12**	0.012878	-6.2789	0.006019	**PCNT**	0.25203	-1.9883	0.04384
**CCDC59**	0.013326	-6.2296	2.72E-05	**FKBP3**	0.25236	-1.9864	0.018169
**LMAN1**	0.027333	-5.1932	0.042953	**MOB1A**	0.2653	-1.9143	0.030963
**APOA1**	0.03057	-5.0317	0.038111	**MOB1B**	0.2653	-1.9143	0.030963
**ADD2**	0.031011	-5.0111	0.01617	**AKR1E2**	0.28381	-1.817	0.032173
**IFIT1B**	0.041147	-4.6031	6.37E-05	**ATAD3A**	0.29107	-1.7806	0.022167
**SNX3**	0.041326	-4.5968	0.024816	**TAGLN2**	0.2965	-1.7539	0.042997
**NUDT5**	0.042438	-4.5585	0.003152	**ISYNA1**	0.2988	-1.7428	0.039036
**PLG**	0.050907	-4.296	0.048898	**UQCRC1**	0.30166	-1.729	0.046911
**STAT5A**	0.058391	-4.0981	0.000126	**PSMD7**	0.3108	-1.6859	0.047249
**STAT5B**	0.058391	-4.0981	0.000126	**PCBP3**	0.32293	-1.6307	0.027612
**ASS1**	0.059334	-4.075	0.014165	**PSMB1**	0.32434	-1.6244	0.019786
**XAB2**	0.070576	-3.8247	9.96E-06	**CASP6**	0.32989	-1.5999	0.018008
**IST1**	0.078729	-3.667	0.002035	**MARS**	0.33485	-1.5784	0.024323
**FLNB**	0.086516	-3.5309	0.049456	**SUMO3**	0.38527	-1.3761	0.047806
**SF3B4**	0.089249	-3.486	0.007161	**SUMO4**	0.38527	-1.3761	0.047806
**RNPS1**	0.091515	-3.4498	0.002526	**PCBP4**	0.39726	-1.3318	0.036537
**GSTM1**	0.10669	-3.2286	0.000517	**OGDHL**	0.39916	-1.325	0.016945
**SMARCA4**	0.10927	-3.1941	0.003828	**BRD3**	0.40584	-1.301	0.044302
**CHMP6**	0.11893	-3.0718	0.038206	**BRD4**	0.40584	-1.301	0.044302
**LONRF1**	0.12849	-2.9603	0.037191	**SEC22B**	0.41374	-1.2732	0.045876
**TROVE2**	0.13048	-2.9381	0.017048	**TMED9**	0.43041	-1.2162	0.047156
**DDX23**	0.14089	-2.8273	0.001712	**U2AF2**	0.43135	-1.2131	0.038516
**RRP9**	0.1425	-2.811	0.032374	**WDR12**	0.49492	-1.0147	0.034212
**UBXN1**	0.14957	-2.7411	0.025959	**RPL29**	0.50423	-0.98785	0.046686
**TFAM**	0.14964	-2.7405	0.017094	**EFTUD2**	0.50881	-0.97481	0.036652
**DES**	0.15359	-2.7029	0.040059	**SEC61B**	0.52215	-0.93746	0.04309
**HSDL2**	0.15747	-2.6668	0.029977	**RPL12**	0.54617	-0.87257	0.039891
**AIFM1**	0.1641	-2.6074	0.047817	**PSMA1**	0.63828	-0.64774	0.000773

Up-regulated proteins							
Protein	FC	log2(FC)	raw.pval	Protein	FC	log2(FC)	raw.pval
ACTN4	1.6847	0.75252	0.03868	NOX3	3.2241	1.6889	0.039892
CAT	1.7882	0.83854	0.018503	BPIFB1	3.39	1.7613	0.045741
XPNPEP3	1.8502	0.88769	0.049443	AIMP1	3.5917	1.8447	0.036606
RPS17	1.9178	0.93945	0.011222	EIF6	3.8201	1.9336	0.036816
ASF1A	1.9986	0.99896	0.038291	HEBP2	3.8631	1.9498	0.02094
RAP1BL	1.9993	0.9995	0.018724	RAD23B	3.8991	1.9631	0.04244
ILF2	2.0133	1.0095	0.043751	FTL	4.0739	2.0264	0.003012
MAT1A	2.0245	1.0176	0.041143	PAFAH1B1	4.1461	2.0518	0.02154
PGAM1	2.1423	1.0991	0.032415	COX6C	4.2019	2.071	0.03068
SLC3A2	2.1581	1.1097	0.023925	CNP	4.6388	2.2138	0.01225
RPS13	2.1598	1.1109	0.019814	SLC1A5	4.765	2.2525	0.009261
PGAM2	2.3031	1.2036	0.048892	RPL19	4.9514	2.3078	0.025304
HNRNPL	2.3048	1.2046	0.032555	KARS	5.011	2.3251	0.018002
STOM	2.3174	1.2125	0.028526	MMP8	5.77	2.5286	0.01831
TUBB8	2.4802	1.3105	0.035731	PRG2	5.7948	2.5347	0.021329
ESYT1	2.5406	1.3452	0.026919	PRTN3	5.9035	2.5616	0.018005
HNRNPA0	2.5433	1.3467	0.029992	EIF5B	5.9663	2.5768	0.020094
HSP90AA5P	2.6262	1.393	0.02647	RPS4X	6.0644	2.6004	0.00343
PRPF8	2.6305	1.3953	0.007932	NACAP1	7.026	2.8127	0.003525
CALR	2.636	1.3983	0.047381	PRG3	7.5801	2.9222	0.047312
CORO1C	2.6907	1.428	0.026591	PDXK	7.6567	2.9367	0.015469
TCEB1	2.72	1.4436	0.027895	RPS4Y1	7.9973	2.9995	0.001841
TMEM14C	2.7436	1.4561	0.036779	RPS4Y2	7.9973	2.9995	0.001841
PSMC6	2.7999	1.4854	0.048488	PSMC3	8.2638	3.0468	0.026073
STOML3	2.8489	1.5104	0.019212	APOH	8.6417	3.1113	0.014398
CREG1	2.9658	1.5684	0.017257	MAPK1	8.9216	3.1573	0.02574
AIMP2	3.103	1.6336	0.02832	MAP7D3	10.399	3.3783	0.001894
ITGAM	3.1149	1.6392	0.024633	SERPINI2	11.668	3.5445	0.014509
PGRMC2	3.2166	1.6855	0.03718	BPGM	20.997	4.3921	0.001353
NOX1	3.2241	1.6889	0.039892	WBP11	159.23	7.315	0.006497

Table S6: List of CD71+ erythroid cells proteins correlated with MELD-Na (severity of disease) . FC represents fold change.

Inversely correlated Proteins with FC value > -0.5					
**RAC2**	-0.5003682	**ERP29**	-0.5302	**YWHAB**	-0.5888
**RAC3**	-0.5003682	**ANK3**	-0.53091	**PFKL**	-0.5892
**SLC25A21**	-0.5007271	**BUD31**	-0.53157	**RAB10**	-0.59316
**HEATR1**	-0.5009556	**ECI1**	-0.53265	**SETDB1**	-0.59341
**S100A11**	-0.5014098	**MAGOHB**	-0.53408	**AKAP8L**	-0.5946
**CETN2**	-0.5015987	**TOMM70A**	-0.53558	**HNRNPH1**	-0.59535
**SCCPDH**	-0.5022743	**PPP2CA**	-0.53574	**HNRNPH2**	-0.59535
**POF1B**	-0.5031412	**PPP2CB**	-0.53574	**TUBA8**	-0.59536
**APOA1**	-0.5064964	**TMED4**	-0.54113	**EPB41**	-0.59766
**TUBA1B**	-0.5065001	**CPVL**	-0.54315	**ALYREF**	-0.59928
**TUBA1A**	-0.5086263	**XAB2**	-0.54327	**STX7**	-0.6008
**TUBA1C**	-0.5099312	**UBXN4**	-0.54524	**P4HB**	-0.60209
**WDR43**	-0.510555	**SPTA1**	-0.5454	**ARPC5**	-0.60276
**MCM4**	-0.5111177	**EZR**	-0.54579	**ASS1**	-0.60576
**PDE4DIP**	-0.5121129	**CYTB**	-0.54646	**IFIT1B**	-0.60684
**SUMO2**	-0.5125212	**MOB1A**	-0.54856	**ARF4**	-0.607
**VIM**	-0.5129027	**MOB1B**	-0.54856	**ANXA1**	-0.60987
**LOC255308**	-0.513249	**TUBA3D**	-0.55039	**TUBA3E**	-0.61026
**PDHA1**	-0.5137413	**SPECC1**	-0.55048	**LDHA**	-0.61289
**ADD2**	-0.5140575	**CLTC**	-0.55116	**AP2A1**	-0.6185
**VDAC3**	-0.5146009	**RPS10**	-0.55117	**NOP58**	-0.61952
**HNRNPD**	-0.5150993	**CAND1**	-0.55141	**ARF1**	-0.62081
**RAB21**	-0.5155706	**TROVE2**	-0.55371	**ARF3**	-0.62081
**LAMTOR3**	-0.5156917	**ACAA2**	-0.55451	**HNRNPM**	-0.62829
**YWHAH**	-0.5168501	**ARPC2**	-0.55491	**TAGLN2**	-0.63304
**ZDBF2**	-0.518325	**TCP1**	-0.55678	**DMTN**	-0.63429
**TSPO**	-0.5196648	**CAPZA1**	-0.56159	**GSTM1**	-0.63501
**ATP6**	-0.5203296	**MINPP1**	-0.56309	**GSTM2**	-0.63501
**ELOVL5**	-0.5206548	**ACADVL**	-0.56312	**GSTM4**	-0.63501
**CRLF3**	-0.521532	**SUMO3**	-0.56362	**GSTM5**	-0.63501
**HSPD1**	-0.5218613	**SUMO4**	-0.56362	**RPL17**	-0.63705
**S100A9**	-0.5249184	**PABPC4**	-0.56375	**TMEM109**	-0.63785
**SEC22B**	-0.5249863	**SNRPA1**	-0.5638	**CCDC59**	-0.63847
**PNPO**	-0.5257395	**SF3B5**	-0.56991	**DAP3**	-0.64036
**MAGT1**	-0.526261	**RTN4**	-0.57083	**HSDL2**	-0.64384
**ACO2**	-0.5267435	**ELAVL1**	-0.57426	**FARSA**	-0.64428
**CLN6**	-0.5272611	**GOLGA4**	-0.57711	**PSME1**	-0.64558
**SAP18**	-0.527846	**CAPN1**	-0.57952	**FKBP8**	-0.64653
**ATP5I**	-0.5290401	**DLST**	-0.58026	**OPTN**	-0.65104
**CSTA**	-0.5301926	**RAB5A**	-0.58131	**ARF5**	-0.65487

Directly correlated Proteins with FC value >0.5					
**HNRNPL**	0.822120036	**CHIT1**	0.661258	**HSPA5**	0.58179
**THYN1**	0.907697148	**APMAP**	0.660274	**RBM25**	0.581776
**NCSTN**	0.811829893	**LAMP1**	0.65875	**MLEC**	0.580405
**SNRNP70**	0.809362692	**PITHD1**	0.656148	**RNASE3**	0.580126
**TMA7**	0.805159219	**NME1**	0.655024	**CTSD**	0.577168
**SIRPA**	0.785866374	**DEFA1**	0.654174	**CAMP**	0.577031
**NPM1**	0.783631072	**PDXK**	0.653735	**CCAR1**	0.576837
**SSBP1**	0.778155888	**GLIPR2**	0.652455	**ARHGDIB**	0.576462
**HNRNPUL1**	0.754318156	**SNRPE**	0.649656	**PPIA**	0.576445
**FUS**	0.750337735	**GUSB**	0.647539	**SMC1A**	0.575042
**TXNL1**	0.750043942	**PLBD1**	0.645433	**HMGB1**	0.572564
**HSPA1B**	0.749246984	**MMP8**	0.645411	**DNAJB11**	0.572404
**RAP1B**	0.742832721	**KARS**	0.645322	**GPX4**	0.571737
**RALA**	0.74235357	**NAA38**	0.638436	**HSP90AA1**	0.571265
**RALB**	0.74235357	**LOC101060545**	0.63584	**BUB3**	0.571244
**HSPA8**	0.741949307	**NCOA5**	0.635664	**PRCP**	0.571021
**NMT2**	0.725450256	**MPO**	0.634992	**LSM2**	0.570288
**SNRPD2**	0.724789346	**ATP2A2**	0.634834	**TRAP1**	0.570251
**RAP1BL**	0.719758561	**AZU1**	0.633246	**ROCK1**	0.569816
**GPD2**	0.716396696	**RHOC**	0.632216	**PPIB**	0.568894
**PRPF40A**	0.715925558	**SERPINB6**	0.631925	**CEACAM8**	0.568376
**EIF5B**	0.715289673	**TMEM205**	0.625215	**SIX5**	0.567683
**SERPINB1**	0.70033458	**HMGB2**	0.623836	**APOD**	0.563151
**MYH9**	0.698749458	**SPTBN1**	0.622425	**TPT1**	0.562372
**IGHG3**	0.696860555	**RHOA**	0.62165	**EEF1G**	0.559561
**CALR**	0.696307597	**RPS27A**	0.621046	**DNAJC7**	0.558873
**GRN**	0.694770224	**UBA52**	0.621046	**SMC3**	0.557935
**APEX1**	0.69363023	**UBB**	0.621046	**HSPA2**	0.557834
**LSM6**	0.680684269	**UBC**	0.621046	**PRG3**	0.557318
**ATP1B3**	0.680209733	**AKR7A2**	0.618329	**IGHA1**	0.556937
**NAGA**	0.675727566	**MTDH**	0.613896	**ITGAM**	0.556715
**IGLC2**	0.675081642	**TKTL2**	0.609686	**ARL8A**	0.556538
**IGLC3**	0.675081642	**LGALS3BP**	0.609472	**TMEM57**	0.556308
**HARS**	0.674997203	**MTCH2**	0.609406	**CAP2**	0.55483
**FLJ44635**	0.665851394	**CST7**	0.609008	**CECR1**	0.553206
**TKT**	0.663923151	**MYH14**	0.601673	**LCN2**	0.550821
**LSM4**	0.663238382	**ELANE**	0.601072	**TIAL1**	0.55

Table S7: Pathway activity scores and statistical comparisons between Control, NCPF, and CLD groups

	Control (Mean ± S.D.)	NCPF (Mean ± S.D.)	CLD (Mean ± S.D.)	Ctrl vs NCPF p value	NCPF vs CLD p value	Ctrl vs CLD p value
**Heme biosynthesis**	0.213 ± 0.20	0.172 ± 0.14	-0.125 ± 0.007	0.7618	0.0240*	0.0481*
**Glutathione activity**	0.009 ± 0.05	0.094 ± 0.15	-0.09 ± 0.14	0.3471	0.1229	0.2595
**Translation**	0.101 ± 0.20	0.098 ± 0.05	-0.066 ± 0.04	0.9704	0.0003**	0.0989
**Cell cycle**	0.002 ± 0.01	0.052 ± 0.19	0.002 ± 0.01	0.5867	0.5867	>0.9999
**Mitochondrial activity**	0.045 ± 0.22	0.109 ± 0.18	-0.056 ± 0.05	0.6714	0.1697	0.4275
**Apoptosis**	0.162 ± 0.19	-0.134 ± 0.09	0.015 ± 0.02	0.0103*	0.0115*	0.1170
**Erythropoiesis**	0.163 ± 0.29	0.222 ± 0.11	-0.133 ± 0.10	0.7670	0.0158*	0.2104

Data are presented as mean ± SD. Statistical significance is indicated as follows: $p < 0.05$ (*), $p < 0.01$ (**), $p < 0.001$ (***), $p < 0.0001$ (****); ns = not significant

Table S8: List of genes with forward and reverse primer sequence.

S.No.	Genes	Forward primer sequence	Reverse primer sequence
1	TFR1	CTGTCCAGCAGCCATAGG	GAACTGCCACACAGAAGAAC
2	TFR2	CTATTCCAGAGAGCGCAACA	GAAGTGGGCCAATGTCTCC
3	IRP1	GTGCAGTCGGAGGAACAC	CCTGGTTGTACAGGATCCAA
4	IRP2	TAATATGGTCTCCGGCGATG	ACCCGTATTGAGTAAGGCAG
5	GATA1	AGTAAACGGGCAGGTACTCA	GGTTCACCTGGTGTAGCTTG
6	KLF1	TGGTTTTCCCACGAATGGAC	TCGGATTTTCCGTAAGAGGC
7	NFE2	TAGCCAGGAAAACAGTTTGGG	ATGGCTCACTTGGAGCATTC
8	LDB1	GCGATGTGAAGATGTCAGTG	ATGTAGGCGGATACATGGGA
9	EIF2A	TTCCAAGGGAATCTGGGAAG	CTTTTTCTCCATTGCCCCAG
10	FAM122A	AAGATGGAGCTAGACCTGGAG	CCGGCGAAGTGTCACTGAG
11	EPOR	CAGCTCCCAGCTCTTG	GGGCACAGTGTCCAT
12	ALAS1	AATCCTTGCTTCAGGGACTC	AGAAGCCACTCATCCATCCA
13	ALAS2	AGGCAAGGTGGTTAAGACTCA	CTCCAGCCTTTGTTGCCTTA
14	Alfa1-globin	TGGACAAGTTCCTGGCTTCT	CCGCCCACTCAGACTTTATT
15	Alfa2-globin	ACCCGGTCAACTTCAAGC	AACGGTATTTGGAGGTCAGC
16	Beta-globin	GCAACCTCAAACAGACACCA	CTCACCACCAACTTCATCCA
17	HMOX1	ATGCCCCAGGATTTGTCA	ACCTGGCCCTTCTGAAAGTT
18	HMOX2	CCACGGCACTTTACTTCACA	CTCCTCCCAGTTTTCACCAA
19	BACH1	AACTGCCATTCAATGCACAA	CAAGTTTTCTCTTGCGACAGC
20	GATA2	CCCAAAGAAGTGTCTCCTGA	GGACTGCCACTTTCCATCTT
21	FLVCR	CTCCCGCATCGCCTCAGTGT	ACTGCAGTTCCAAGCTGATT

Table S9: List of Real time PCR fold change (FC) values of different genes in cirrhosis. FC represents fold change.

Gene's Name	Fold change (FC)
**Alfa1-globin**	0.373194596
**Alfa2-globin**	2.210695005
**Beta-globin**	0.126262664
**ALAS1**	0.158713857
**ALAS2**	0.539240216
**HMOX1**	0.057074891
**HMOX2**	0.249394231
**EPOR**	0.517811891
**BACH1**	0.686818117
**GATA2**	1.008701984
**FLVCR**	0.963595793
**TFR1**	0.197031746
**TFR2**	1.491744027
**IRP1**	0.579748867
**GATA1**	0.233096622
**KLF1**	1.110338834
**LDB1**	0.615145672
**IRP2**	2.740510086
**EIF2ALFA**	0.846093431
**NFR2**	1.41966994
**NFE2**	4
**FAM122**	0.53547417

Table S10: List of 26 erythropoiesis regulating factors or cytokines or growth factors in control (n=3), NCPF (n=7), cirrhosis (n=60). Among cirrhosis, alcoholics (n=30) & NASH (n=30). p-value significance <0.05.

Cytokines (Average ± Standard Error)							
S.N.	Cytokines/ Growth factors	Control	NCPF	Cirrhosis			p value
				All	Alcoholic	NASH	
	Erythropoiesis regulating factors						
1	**IL-3**	103 ± 2.6	89.1 ± 10.5	**102 ± 4.7**	110 ± 7.8	93.4 ± 4.5	0.064
2	**IL-6**	36.6 ± 1.7	34.7 ± 3.02	**52.05 ± 4.9**	47 ± 4.56	56.8 ± 8.7	0.327
3	**FLT3**	198 ± 19.6	175 ± 25	**199 ± 21.6**	179 ± 12.7	218 ± 41.4	0.386
4	**SCF**	91.5 ± 5.25	125 ± 19.2	**101 ± 5.5**	94.6 ± 6.7	108 ± 8.7	0.213
5	**EPO**	8.2 ± 0.7	13.69 ± 2.6	**27.3 ± 4.9**	14.7 ± 4.8	39.9 ± 6.8	**0.008****
6	**TPO**	91.3 ± 3.13	66.5 ± 7.05	71 ± 3.5	76.8 ± 4.6	65.3 ± 5.3	0.107
7	**Hepcidin**	3.43 ± 0.47	7.09 ± 1.4	13.3 ± 1.42	15.38 ± 2.18	11.4 ± 1.76	0.172
8	**Ferritin**	18282 ± 1360	13195 ± 3508	12857 ± 1071	14658 ± 1447	11056 ± 1525	0.093
9	**Transferrin**	62.2±15.8	64.13±14.7	**118.6 ± 61.7**	140.7 ± 72.8	94.7± 34	**0.006****
	Inflammatory cytokines						
1	**IL-1α**	41 ± 2.1	32.7 ± 4.02	35.6 ± 1.19	37 ± 1.61	33.6 ± 1.7	0.123
2	**IL-1β**	44.75 ± 3.1	33.6 ± 7.9	36.7 ± 2.5	38.9 ± 4.09	34.4 ± 3.05	0.387
3	**IL-2**	34 ± 0.83	27.9 ± 3.9	29.7 ± 1.2	32 ± 1.56	27 ± 1.75	**0.041***
4	**IL-5**	11.25 ± 0.4	10.2 ± 0.52	**12.1 ± 0.4**	12.7 ± 0.6	11.5 ± 0.59	0.166
5	**IL-12**	45 ± 1.95	31.5 ± 5.22	**34.6 ± 1.6**	37 ± 2.3	31.6 ± 2.2	0.079
6	**IFN-γ**	45.6 ± 1.2	33.7 ± 3.5	45.9 ± 3.4	55 ± 5.8	36.5 ± 2.5	0.007
7	**TNF-α**	48.3 ± 4.4	43.2 ± 5.6	40.9 ± 2.3	45.3 ± 3.4	36.2 ± 2.75	**0.044***
8	**CCL2/MCP1**	92.8 ± 24	159 ± 18.7	97.2 ± 12.4	102 ± 15.3	91 ± 20.3	0.656
9	**S100A9**	2826 ± 691	27.3 ± 328	3451 ± 238	3731 ± 386	3162 ± 271	0.234
10	**TRAIL-R2**	106 ± 3.9	128 ± 14.1	**217 ± 13.5**	237 ± 21.4	194 ± 15.06	0.105
	Anti-inflammatory cytokines						
1	**IL-1RA**	2551 ± 323	1373 ± 155	**1943 ± 126**	2049 ± 162	1830 ± 195	0.394
2	**IL-4**	77.6 ± 3.47	69 ± 18.8	**67.9 ± 5.2**	74.3 ± 7.8	61 ± 6.7	0.209
3	**IL-10**	56.6 ± 4.9	35.07 ± 4.1	**38.7 ± 2.9**	41.7 ± 4.47	35.5 ± 3.86	0.294
4	**TGF-α**	153 ± 7.06	183 ± 30.7	309 ± 25	334 ± 35	282 ± 37.2	0.311
5	**TGF-β**	25 ± 3.12	24.4 ± 4.1	56 ± 6.57	78.6 ± 10.6	33.95 ± 4.6	**0.0005*****
6	**LIF**	33 ± 1.24	25.07 ± 3.6	**26.9 ± 1.7**	29.8 ± 2.96	23.8 ± 1.55	0.077
7	**G-CSF**	52.3 ± 2.69	32.7 ± 5.69	**37.6 ± 2.18**	40.3 ± 3.02	34.7 ± 3.12	0.202

Data are presented as mean ± SD. Statistical significance is indicated as follows: $p < 0.05$ (*), $p < 0.01$ (**), $p < 0.001$ (***), $p < 0.0001$ (****); ns = not significant

Table S11: Correlation of 23 cytokines or growth factors with hemoglobin.

S.No.	Cytokines	Pearson Correlation	Sig. (2-tailed)
1	**TNF-α**	-0.067	0.601
**2**	**IL-6**	**-0.255**	**0.044***
3	**IL-3**	0.161	0.206
4	**Ferritin**	-0.049	0.703
5	**IL-10**	-0.073	0.568
6	**CCl2/JE/MCP1**	-0.086	0.504
7	**SCF**	-0.074	0.562
8	**IL-1β**	0.043	0.74
9	**IFN-γ**	0.046	0.719
10	**IL-1RA**	-0.222	0.08
11	**LIF**	0.024	0.855
12	**IL-1A**	-0.073	0.572
13	**IL-4**	0.145	0.258
14	**IL-2**	-0.001	0.995
15	**TRAIL-R2**	-0.188	0.141
16	**S100A9**	-0.029	0.819
17	**IL-5**	-0.076	0.556
**18**	**GCSF**	**-0.293**	**0.02***
19	**TPO**	0.145	0.257
20	**IL-12**	-0.053	0.68
21	**TGF-α**	-0.046	0.721
22	**GM-CSF**	0.015	0.907
23	**FLT3**	-0.029	0.819

Statistical significance is indicated as follows: $p < 0.05$ (*), $p < 0.01$ (**), $p < 0.001$ (***), $p < 0.0001$ (****).

Table S12: Spearman correlation of significant cytokines with ROS.

	ROS vs. IL-6	ROS vs. IL-3	ROS vs. IL-10	ROS vs. TRAIL R2	ROS vs. IL-5	ROS vs. GCSF	ROS vs. TFR	ROS vs. SCF
Pearson r								
r	-0.2752	0.9941	0.2382	-0.2031	-0.6841	0.767	0.256	-0.8337
95% confidence interval	-0.9313 to 0.8017	0.9091 to 0.9996	-0.8154 to 0.9259	-0.9204 to 0.8274	-0.9768 to 0.4999	-0.3566 to 0.9836	-0.8090 to 0.9285	-0.9887 to 0.1837
R squared	0.07575	0.9882	0.05672	0.04125	0.468	0.5882	0.06553	0.6951
P (two-tailed)	0.6541	0.0005	0.6997	0.7432	0.2027	0.1302	0.6777	0.0793
P value summary	ns	***	ns	ns	ns	ns	ns	ns
Significant? (alpha = 0.05)	No	Yes	No	No	No	No	No	No

Statistical significance is indicated as follows: $p < 0.05$ (*), $p < 0.01$ (**), $p < 0.001$ (***), $p < 0.0001$ (****); ns = not significant
